# Who pays to treat malaria and how much? Analysis of the cost of illness, equity and economic burden of malaria in Uganda

**DOI:** 10.1093/heapol/czae093

**Published:** 2024-10-15

**Authors:** Katherine Snyman, Catherine Pitt, Angelo Aturia, Joyce Aber, Samuel Gonahasa, Jane Frances Namuganga, Joaniter Nankabirwa, Emmanuel Arinaitwe, Catherine Maiteki-Sebuguzi, Henry Katamba, Jimmy Opigo, Fred Matovu, Grant Dorsey, Moses R Kamya, Walter Ochieng, Sarah G Staedke

**Affiliations:** Infectious Diseases Research Collaboration (IDRC), Plot 2C Nakasero Road, Kampala P.O. Box 7475, Uganda; Department of Global Health & Development, London School of Hygiene & Tropical Medicine (LSHTM), Keppel Street, London WC1E 7HT, United Kingdom; Department of Global Health & Development, London School of Hygiene & Tropical Medicine (LSHTM), Keppel Street, London WC1E 7HT, United Kingdom; Infectious Diseases Research Collaboration (IDRC), Plot 2C Nakasero Road, Kampala P.O. Box 7475, Uganda; Infectious Diseases Research Collaboration (IDRC), Plot 2C Nakasero Road, Kampala P.O. Box 7475, Uganda; Infectious Diseases Research Collaboration (IDRC), Plot 2C Nakasero Road, Kampala P.O. Box 7475, Uganda; Infectious Diseases Research Collaboration (IDRC), Plot 2C Nakasero Road, Kampala P.O. Box 7475, Uganda; Infectious Diseases Research Collaboration (IDRC), Plot 2C Nakasero Road, Kampala P.O. Box 7475, Uganda; Infectious Diseases Research Collaboration (IDRC), Plot 2C Nakasero Road, Kampala P.O. Box 7475, Uganda; Infectious Diseases Research Collaboration (IDRC), Plot 2C Nakasero Road, Kampala P.O. Box 7475, Uganda; National Malaria Control Programme, Ministry of Health (MOH/NMCP), Plot 6 Lourdel Rd, Nakasero, Kampala, Uganda; National Malaria Control Programme, Ministry of Health (MOH/NMCP), Plot 6 Lourdel Rd, Nakasero, Kampala, Uganda; National Malaria Control Programme, Ministry of Health (MOH/NMCP), Plot 6 Lourdel Rd, Nakasero, Kampala, Uganda; School of Economics, Makerere University, Plot 51, Pool Road, Kampala, Uganda; University of California, San Francisco (UCSF), 1001 Potrero Avenue, San Francisco, CA 94110, United States; Infectious Diseases Research Collaboration (IDRC), Plot 2C Nakasero Road, Kampala P.O. Box 7475, Uganda; Department of Medicine, Makerere University, New Mulago Hill Road, Mulago, Kampala, Uganda; Centers for Disease Control and Prevention, 1600 Clifton Rd, Atlanta 30329, Georgia, Georgia; Infectious Diseases Research Collaboration (IDRC), Plot 2C Nakasero Road, Kampala P.O. Box 7475, Uganda; Liverpool School of Tropical Medicine, Pembroke Place, Liverpool L3 5QA, United Kingdom

**Keywords:** Cost-of-illness, malaria, out-of-pocket expenditure, economic burden, equity

## Abstract

Case management of malaria in Africa has evolved markedly over the past 20 years and updated cost estimates are needed to guide malaria control policies. We estimated the cost of malaria illness to households and the public health service and assessed the equity of these costs in Uganda. From December 2021 to May 2022, we conducted a costing exercise in eight government-run health centres covering seven sub-regions, collecting health service costs from patient observations, records review and a time-and-motion study. From November 2021 to January 2022, we gathered data on households’ cost of illness from randomly selected households for 614 residents with suspected malaria. Societal costs of illness were estimated and combined with secondary data sources to estimate the total economic burden of malaria in Uganda. We used regression analyses and concentration curves to assess the equity of household costs across age, geographic location and socio-economic status. The mean societal economic cost of treating suspected malaria was $15.12 [95% confidence interval (CI): 12.83–17.14] per outpatient and $27.21 (95% CI: 20.43–33.99) per inpatient case. Households incurred 81% of outpatient and 72% of inpatient costs. Households bore nearly equal costs of illness, regardless of socio-economic status. A case of malaria cost households in the lowest quintile 26% of per capita monthly consumption, while a malaria case only cost households in the highest quintile 8%. We estimated the societal cost of malaria treatment in Uganda was $577 million (range: $302 million–1.09 billion) in 2021. The cost of malaria remains high in Uganda. Households bear the major burden of these costs. Poorer and richer households incur the same costs per case; this distribution is equal, but not equitable. These results can be applied to parameterize future economic evaluations of malaria control interventions and to evaluate the impact of malaria on Ugandan society, informing resource allocations in malaria prevention.

Key messages

- Point 1: Malaria illness is costly, with households bearing ≥70% of costs. Productivity losses drive costs, and these estimates are sensitive to methodological choices particularly how time is valued.
- Point 2: The richest households pay slightly higher costs than the poorest, which is not proportional to their consumption.
- Point 3: The societal impact of malaria treatment in Uganda is high, costing $577 million (1.4% GDP), suggesting investment in prevention is needed.

## Introduction

Malaria remains a major public health problem, with negative social and economic consequences in endemic areas (World Health Organization, 2022). The burden of malaria is unevenly distributed and is disproportionately concentrated among pregnant women and children living in sub-Saharan Africa (World Health Organization, 2022). The association between malaria and poverty is well-described, although the causal pathways between them are complex and less understood (Tusting *et al*., 2013; Sarma *et al*., 2019). The costs of controlling and treating malaria can strain health systems and economies, especially where resources are limited. These costs can further impoverish households (Alam and Mahal, 2014).

Despite intensified efforts to control malaria over the past 15 years, the burden of malaria in Uganda remains high, and is up to 10 times higher among the poorest individuals compared to the wealthiest (Ugandan Bureau of Statistics, 2018; World Health Organization, 2022). Access to prompt and appropriate malaria treatment is more common for rural children (Humphreys *et al*., 2021) and children from wealthier households (Evans *et al*., 2019). Yearly investment in malaria control in Uganda has ranged between $115 and $160 million US dollars (USD) per year since 2012, with >90% of funding coming from external donors including The Global Fund and the US President’s Malaria Initiative (The Global Fund, 2020). Insufficient funding combined with a high burden of malaria necessitates the efficient allocation of resources in malaria control programmes (Scott *et al*., 2017). Policymakers often require evidence on the costs, economic and equity impacts of interventions for decision-making.

Since 2019, three systematic reviews, applying different methods, have examined the cost of malaria illness in various contexts and populations (El-Houderi *et al*., 2019; Conteh *et al*., 2021; Andrade *et al*., 2022). These reviews found substantial heterogeneity in the cost of treating uncomplicated and severe malaria cases and in the costs to providers and households. This heterogeneity has limited the generalizability of the estimates. These reviews did not examine equity.

The World Health Organization’s CHOosing Interventions that are Cost-Effective programme (WHO-CHOICE) provides standard estimates of the cost of inpatient and outpatient visits by country, which are widely used to estimate cost savings from malaria cases averted (Morel *et al*., 2005, Mangham, 2009a). However, these estimates were last updated in 2010, are not necessarily generalizable to rural settings, and do not account for variation between the costs of malaria and non-malaria visits (Gkountouras *et al*., 2011; World Health Organization, 2011). More current estimates are therefore needed to guide malaria control policies.

In 2003, the economic loss attributed to malaria morbidity in Uganda was estimated at $49 million USD ($2 per capita) (Orem *et al*., 2012). However, the epidemiology and economics of malaria has changed, and these estimates are outdated (Rosenthal, 2022). Many studies have estimated the economic cost of malaria treatment in Uganda. Some used data collected before 2011 (Lubel *et al*., 2010; Batwala *et al*., 2011; Orem *et al*., 2011; 2012; Matovu *et al*., 2014; Menon *et al*., 2016). Others focused on particular aspects including case management(Batwala *et al*., 2011), home-based management (Lubel *et al*., 2010), community health workers (Hansen *et al*., 2017b), out-of-pocket (OOP) costs (Menon *et al*., 2016), household costs (Matovu *et al*., 2014) or the private sector (Hansen *et al*., 2017a).

Health care financing in Uganda has also changed over the last 15 years (Kwesiga *et al*., 2020; Ssennyonjo *et al*., 2021). Malaria case management has also been transformed by increased availability and reduced cost of diagnostics and artemisinin-based treatments (Kibira *et al*., 2021). These older cost estimates may not reflect the current burden to households or health service.

To address these evidence gaps, we aimed to estimate the cost of malaria illness to the health service and households in Uganda, and to assess the equity in the distribution of these costs. First, we estimated the cost per outpatient and per inpatient case from a disaggregated societal perspective. Second, we investigated how the cost per case varies by equity-relevant variables such as household geographic location and socio-economic status, and the age and gender of the patient. Third, we used the estimated cost per episode to calculate the societal cost of malaria illness for the whole of Uganda in 2021. Our estimates are designed to support future malaria prevention studies in Uganda, quantify the economic burden of malaria on Ugandan society and inform investment priorities.

## Methods

### Study setting

In 2021, the population of Uganda was 41 million, the GDP per capita was $884 USD, and 4% of GDP was spent on health (Ugandan Bureau of Statistics, 2021; World Bank 2023b). Malaria is endemic in 95% of the country (Ugandan Ministry of Health, 2021). Uganda’s Malaria National Strategic Plan focuses on provision of long-lasting insecticide-treated nets (LLINs), with mass distribution campaigns carried out every 3–4 years; targeted indoor residual spraying of insecticides; case management with artemisinin-based combination therapies (ACTs); health promotion messaging; and intensified malaria surveillance (Ugandan Ministry of Health, 2014).

Uganda is divided into 4 regions, 15 sub-regions, 146 districts, 322 counties and 1488 sub-counties (Ugandan Bureau of Statistics, 2023). Uganda’s government-run health service consists of national, regional and general (district) hospitals, and health centres providing four levels of care. Level I health centres (HCI) comprise village health teams primarily offering preventive services; Level II health centres (HCII) provide outpatient services, serving a population of approximately 5000 people; Level III health centres (HCIII) provide outpatient and some inpatient services, serving a sub-county with approximately 20 000 people; and Level IV health centres (HCIV) typically have a laboratory and offer surgical services and blood transfusions, serving a county with approximately 100 000 people (Ugandan Ministry of Health, 2019). Health centres are staffed by a facility in-charge (medical or clinical officer), clinical officers, nurses and laboratory technicians, supported by unpaid community volunteers. A HCIII typically has one clinical officer, while a HCIV has several (Tashobya *et al*., 2006).

Health centres are financed directly by the Ministry of Health or through donations from various non-governmental organizations. Officially, services are provided free of charge, although informal payments for services and medications are widely reported (Ugandan Bureau of Statistics, 2021).

### Study overview

**Figure 1.**
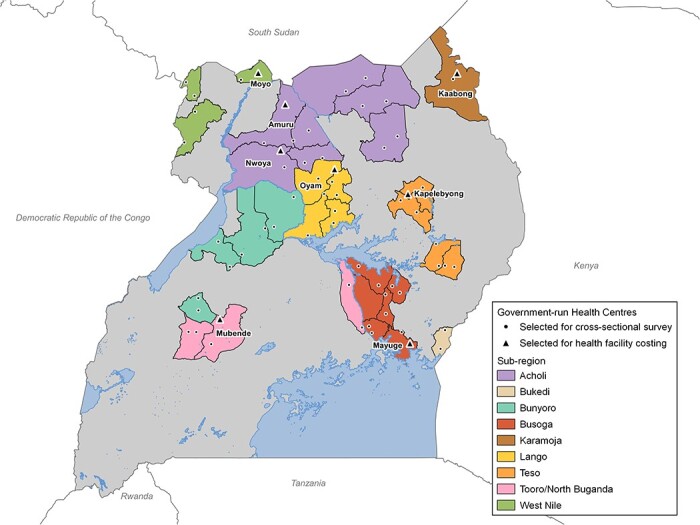
Map of Uganda and study sites

This study was embedded in a large-scale, cluster-randomized trial—the Long-Lasting Insecticidal Nets Evaluation Uganda Project-2 (LLINEUP2)—designed to evaluate the impact of LLINs delivered in 2020–2021 through a mass distribution campaign (Okiring *et al*., 2022). LLINEUP2 was conducted in 64 clusters from 32 districts with intense malaria transmission (Figure 1). Clusters were defined as target communities (1–7 villages) surrounding selected government-run health centres (HCIII or HCIV) with enhanced malaria surveillance provided by the LLINEUP2 study. We employed a disaggregated, societal perspective to this analysis; estimates include costs to the health service and households, separately and combined (Wilkinson *et al*., 2016). The health service perspective combines domestically generated resources from the Ministry of Health and donated items and funds for the provision of health services to the public. The household perspective includes any OOP payments incurred by the household and productivity losses from household members. For health service cost estimates, we defined a malaria case based on clinical diagnoses from the outpatient and inpatient department registers. For household and societal cost estimates, we define a suspected malaria case as a febrile episode and a confirmed malaria case as a case parasitologically confirmed with a diagnostic test. Consistent with recent systematic reviews (El-Houderi *et al*., 2019; Conteh *et al*., 2021), we assumed that malaria cases treated as inpatients (staying at least one night at a facility) were severe malaria, and all other malaria cases—whether treated as outpatients or not treated—were uncomplicated.

**Figure 2.**
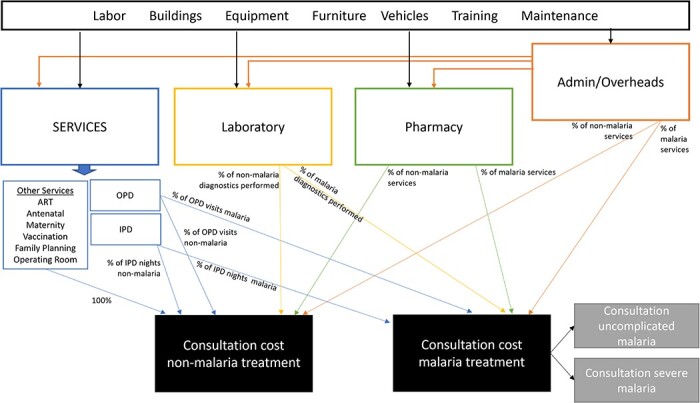
Step-down costing methodology used to allocate provider consultation costs to malaria services

We collected detailed data on capital and recurrent resource use and costs to the health service from December 2021 to May 2022 at eight of these health centres. Capital, labour, training and maintenance costs were allocated using step-down methods (Figure 2). Consumables were estimated using a combination of step-down and micro-costing methods. We collected data on costs to the household via cross-sectional community surveys of households from all 64 clusters in November 2021 and January 2022. Where possible, resource use and price data were collected separately. All costs were collected in Ugandan shillings (UGX). Our economic burden analysis assumed a counterfactual scenario where no malaria cases occur and utilized an incidence-based approach to estimate the potential costs that could be averted if all new cases were prevented (Hodgson and Meiners, 1982). A cost-of-illness evaluation checklist was used (Supplementary Materials) (Larg and Moss, 2011).

### Health centre data collection

Eight health centres were randomly chosen from the 64 health centres participating in LLINEUP2 (Figure 1). We collected resource use and cost data from six HCIIIs and two HCIVs located in seven sub-regions across Uganda. Two researchers spent a week at each health centre, where they reviewed records (expenditure records, staff rosters including salary designations, laboratory registries, pharmacy registries, supply and medicine and supply delivery notes and final services registers); inventoried all capital goods and interviewed staff to collect data from the July 2020-June 2021 financial year.

We also observed several inpatients with clinically diagnosed malaria and recorded the medicines and medical supplies used. We conducted a time-and-motion study to assess the fraction of health workers’ time spent on malaria and non-malaria case management (Lopetegui *et al*., 2014). At each health centre, we observed staff treat consecutive outpatients, including the initial consultation and any follow-up visits that day, until a minimum of 10 clinically diagnosed malaria and 10 non-malaria cases were captured (*n* = 160 in total). We also observed malaria diagnostic testing in the laboratory and interviewed staff to identify the consumables used.

### Cross-sectional household survey

A cross-sectional survey was conducted in the 64 communities participating in LLINEUP2. All households within the target areas were mapped and enumerated to generate a sampling frame for community surveys. Households were randomly selected from the enumeration lists for each cluster and screened until 50 households with at least one child aged 2–10 years were enrolled. Households were included if at least one adult >18 years old was present, usually resident, slept in the household on the night before the survey, and provided informed consent. Households were excluded if the house had been destroyed or could not be found, the house was vacant, or no adult resident was home on at least four occasions.

A questionnaire was administered to the head of the household (or their designate) to gather information on household characteristics, residents and proxy indicators of wealth including asset ownership. Household heads were interviewed about fever treatment sought over the past 14 days for all household members including OOP costs for consultation, diagnosis, medicines, transport and food and time lost due to caregiving or illness (Hansen and Yeung, 2009). Care-seeking costs were collected up to the first two sources of care outside of the home. We collected data on OOP cost by asking how much household members paid OOP at a given place of care in total (Method 1), and then asking more detailed, disaggregated questions for each cost category (Method 2) (Agorinya *et al*., 2021).

### Data analysis

#### Analysis overview

We estimated financial costs, comprising resources that are paid for, and economic costs which reflect the full value of resources used, including those that do not incur a financial cost such as donated funds, goods, services or time (Drummond *et al*., 2015). Household financial cost estimates comprised OOP costs while household economic cost estimates also include productivity losses. Financial and economic costs were estimated overall and disaggregated by facility type (government-run vs private) and site of treatment (outpatient vs inpatient).

We disaggregated our results by care-seeking, i.e. individual with suspected malaria who did not seek care, those who sought care and those with confirmed malaria. Mortality-related costs were only included in the national burden estimate. We did not estimate costs related to long-term sequalae of malaria. We analysed data using Microsoft Excel and STATA 14 (StataCorp, 2015). We inflated all costs to 2022 values using GDP deflators and then converted to 2022 USD using the mean exchange rate ($1 = 3691 UGX) (International Monetary Fund, 2023; ‘USD to UGX Exchange Rate History for 2022, 2022’). Primary results are presented in the paper and further results and data analysis methods can be found in the Supplementary Material.

#### Health service costs

Health centre costs were categorized as labour, capital (buildings, equipment, furniture, vehicles), overheads (training, maintenance, other), diagnostics, medicines or supplies. We estimated the costs of consultations and care separately from the costs of consumables, before combining them into a total cost per outpatient and inpatient case for each health centre.

For the cost of consultation and care (labour, capital and overheads), we used step-down costing methods to allocate resources across all health facility outputs (Figure 2). We used *t*-tests to check for differences in mean consultation time between clinically diagnosed malaria and non-malaria outpatients within each facility observed during the time-and-motion study. We assessed variation in visit time across the eight health centres using one-way analysis of variance. Using data from the registers, we calculated the percentage of all outpatient visits and inpatient nights that were clinically diagnosed as malaria cases to allocate staff time, space, capital costs, overhead costs and diagnostic services. All capital costs were annualized over the useful life of the asset and discounted at a rate of 3%, consistent with the iDSI reference case (Wilkinson *et al*., 2016). We categorized the value of time of paid health service staff as financial costs and community volunteers as additional economic costs. We also estimated labour costs for inpatients using micro-costing methods to compare to our top-down estimates. Wage scales were obtained from national public records (Ugandan Ministry of Public Service, 2020).

We estimated costs of consumables (medicines, diagnostic tests, medical supplies) using a combination of step-down and micro-costing methods. We used reference pricing (The Global Fund, 2022a; 2022b) for rapid diagnostic tests (RDTs) and antimalarial medicines plus shipping and used health centre delivery receipts to obtain all other consumable prices. We calculated consumables cost per RDT and microscopy test performed. Based on observations, we assumed inpatient and outpatient malaria cases had approximately equal diagnostics resource use. To assess variation in health service cost per case between the health centres, we used *t*-test for difference of two means and Pearson’s product–moment correlation as appropriate.

#### Household costs

We estimated the household cost of illness for all suspected malaria cases. For OOP costs, there was a strong positive correlation between costs collected via Method 1 (single question) and Method 2 (multiple questions) (*r* = 0.74; *P* < .001) and Method 1 was on average higher ($1.13 vs $0.97). We used OOP cost estimates from Method 1 in the main analysis, and the detailed cost data collected using Method 2 to assess cost drivers. Productivity losses for those aged ≥12 years were estimated using the human capital approach; the same value for time lost by different individuals was assigned, regardless of occupation (Hodgson and Meiners, 1982; Hansen and Yeung, 2009).

We assumed a 22-day work month and used mean monthly household consumption expenditure for rural households divided by the mean observed number of adults per household in the study (*n* = 2.07) as a proxy for a daily loss of productivity (12 141 UGX = $3.29 USD) (Ugandan Bureau of Statistics, 2021). We assumed 100% productivity losses if residents reported missing work due to illness or caregiving and 50% productivity losses if the resident reported illness but did not report missing work. If a respondent was still ill at the time of the survey, we used the mean illness duration for those respondents who had recovered to project the expected duration of illness.

#### Societal costs

We estimated a societal mean cost per case of malaria combining clinically diagnosed malaria health service costs with suspected malaria household costs. For residents who did not seek treatment, only productivity losses were captured. For residents who sought care at government-run health centres, we avoided double-counting by first attributing the OOP costs to the household and then attributing to the health service only the cost of diagnostics and medicines in excess of the OOP cost (if any). To assess parameter uncertainty and identify which parameters were most influential in societal cost estimates, we used univariate deterministic sensitivity analysis of key input variables. We performed analyses separately for cost per outpatient and inpatient case and reported sensitivity analyses in tornado diagrams.

#### Equity analysis

We sought to understand the equity of the distribution of household costs across households by exploring whether equity-relevant factors were associated with the variation in treatment-seeking behaviours and costs incurred by households (O’Donnell *et al*., 2007). Informed by global guidance (Mangham, 2009b; Cochrane, 2024; World Health Organization, 2024), and previous research, we a priori hypothesized that equity-relevant factors, including age (Sicuri *et al*., 2011), gender (Bates *et al*., 2004), education (Malaney *et al*., 2004), relation to the household head, household wealth (Onwujekwe *et al*., 2010) and geographic location (Tusting *et al*., 2016), could be associated with variations in treatment-seeking behaviour and healthcare costs. We split age into two categories based on differences in health burden (Carneiro *et al*., 2010).

We constructed a study wealth index using principal components analysis, excluding any variables related to household construction which can be associated directly with malaria and increase the association between socio-economic status and malaria outcomes (Vyas and Kumaranayake, 2006; Tusting *et al*., 2016). The wealth index was used to categorize households from poorest to wealthiest. We generated two variables, study wealth quintiles for descriptive tables and study wealth percentiles (a continuous variable) for regression analyses. We used EquityTool to assign the respondents in our study population to Uganda-wide national wealth quintiles (Metrics for Management, 2022). To understand the relative financial impact on households, we compared estimates for the cost per malaria case treated in our study with these estimates of consumption expenditure by national quintile (World Bank, 2023a). First, we examined how household costs varied by individual equity-relevant variables, using *t*-tests for a difference of means and Pearson’s product–moment correlation tests, as appropriate. To describe variation in household costs by wealth index, we produced concentration curves and indices with wealth index as the ranking variable (Erreygers and Van Ourti, 2011; O’Donnell *et al*., 2016).

We then performed a multivariable analysis to identify key equity-relevant factors associated with variation in household cost of malaria illness, including variables selected a priori (Sun *et al*., 1996). We removed outliers and constructed age, gender, head of household as binary variables, sub-region as a categorical variable and wealth as a continuous variable. Our dependent variable is highly skewed with a large mass of zeros, which we consider to be true zeros, so we used a two-part model; the first part estimates a logit model using the full sample and gives probability that a person has any illness costs, and the second part estimates a generalized linear model on the subset of people who had any illness costs (Mihaylova *et al*., 2011). We used a Box-Cox test to choose the natural log-link function (*δ *= 0.12) and the modified Park test to choose a Gamma distribution (coeff.: 2.0). To find a parsimonious model, we started by including all independent variables in the selection equation and primary equation. We applied stepwise backward elimination, using likelihood ratio tests to determine whether to remove variables from the model. In each iteration, we removed the variable with the highest *P*-value. The elimination process continued until the selection parameter reached 0.05 (Royston and Sauerbrei, 2008). We included age as a linear variable during robustness checks and saw no difference. We performed goodness-of-fit tests (Pearson’s correlation, Pregibon link and modified Hosmer–Lemeshow tests) to ensure correct model specification (Belotti *et al*., 2015).

#### Analysis of economic burden

We estimated the total societal cost of malaria illness in Uganda, disaggregated by health service and household perspectives. The annual number of malaria cases, deaths and treatment rates were taken from the World Malaria Report (World Health Organization, 2022). Based on expert opinion at the Ugandan Ministry of Health and WHO assumptions, we assumed that 1–5% of uncomplicated malaria cases progressed to severe illness, 50–80% of severe cases were hospitalized (World Health Organization, 2022) and treatment-seeking rates were the same across the population. For mortality-related productivity losses, we estimated a net present value of lost productivity using the human capital approach, assuming a 3% discount rate and using non-health expenditure GDP per capita over the lost working year. We assumed the average age at death due to malaria was 5 years, with a life expectancy of 65 years, 58 (age 12–65 years) of which would be working years (World Health Organization, 2020; Oumo *et al*., 2022).

## Results

### Health service perspective

From July 2020 to June 2021, health centres recorded a mean of 7185 (median: 7095; range: 2328–11 237) clinically diagnosed malaria outpatient visits and 407 (median: 424; range: 26–1063) inpatient admissions for a mean duration of 2.3 nights (range: 1.0–3.4) (Table 1). We observed 279 outpatient consultations, including 126 clinically diagnosed malaria and 153 non-malaria cases. The time health workers spent on a consultation did not differ significantly between malaria and non-malaria cases overall [3.8 vs 3.6 min; difference 0.1 min; 95% confidence interval (CI): −0.52 to 0.74; *P* = 0.72], or at any individual health centre. Consultation time did not differ significantly between the health centres, except for Health Centre IV, where consultation time was significantly longer (Table 1).

**Table 1.** Descriptive statistics and time and motion results from health centres

Health centre number	1	2	3	4	5	6	7	8	
Characteristics of health centres									
District	Kapelebyong	Kaabong	Oyam	Nwoya	Moyo	Amuru	Mubende	Mayuge	
Sub-region	Teso	Karamoja	Lango	Acholi	West Nile	Acholi	Nort Buganda	Busoga	
Region	East	North	North	North	North	North	Central	East	
Health centre level ^a^	III	III	III	III	III	IV	III	IV	Total
Total outpatient malaria cases ^b^	7605	6584	9099	6355	5954	11 237	2328	8316	57 478
Total inpatient malaria admissions (% total admissions) ^b^	576 (47%)	143 (15%)	519 (23%)	26 (35%)	362 (74%)	1063 (61%)	82 (47%)	486 (60%)	3257 (45%)
Total inpatient nights for malaria (% of total nights) ^b^	1416 (59%)	358 (22%)	970 (15%)	89 (57%)	530 (28%)	2765 (39%)	259 (47%)	2081 (25%)	859 (34%)
Mean nights per malaria admission	2.5	2.5	1.9	3.4	1.5	2.6	3.2	1	2.3
Total RDTs performed	11 019	11 735	8608	11 137	6717	15 150	3507	10 671	78 544
Total microscopy performed	5322	239	4941	1376	1662	282	2062	3729	19 613
Time & motion observations									
Number of malaria outpatient consultations observed	17	17	27	7	19	10	17	12	126
Number of non-malaria outpatient consultations observed	12	42	14	25	20	7	22	11	153
Mean malaria consultation time (min)	3.5	3.5	3.8	10.2	2.7	4.1	3.2	3.1	3.8
Mean non-malaria consultation time (min)	2.9	3.1	3.7	5.8	3	3.6	2.9	3.4	3.6

a Health centres are categorized I–V at the district level in Uganda based on services offered, with a Health Centre I offering the least number of services. Generally, Health Centre IV have all the same services as a Health Centre III, with the addition of surgery. (Ugandan Ministry of Health, 2019).

b Total outpatient cases and inpatient cases and night recorded from July 2020—June 2021.

The mean health service costs were more than three times higher for an inpatient malaria case than for an outpatient case both in terms of financial costs ($19.77 vs $5.84) and economic costs ($21.84 vs $6.78) (Table 2). Volunteers were the main donated resource, driving the difference between economic and financial costs. Drivers of economic costs for outpatient care included labour (64%), diagnostics (16%) and medicines (12%) (Table 2), while cost drivers for inpatient care included labour (47%), medicines (25%) and medical supplies (14%). Medicines used were six times more costly for inpatients than for outpatients ($5.45 vs $0.81). Top-down methods produced a higher estimate of labour costs (excluding administration labour) per inpatient case than bottom-up methods ($7.89 vs $4.32), suggesting that bottom-up methods could underestimate, or top-down methods can overestimate costs.

**Table 2.** Financial and economic cost per clinically diagnosed malaria episode, health service perspective

**Cost category**		**Outpatient cases**		**Inpatient cases**	
**Consultation & care costs**		**Financial (range^c^**)	**Economic (range^c^**)	**Financial (range ^c^**)	**Economic (range^c^**)
Recurrent costs	Labour	3.49 (2.49–3.99)	4.37 (3.46–5.27)	8.22 (2.49–13.30)	10.22 (3.75–16.67)
	Overheads^a^	0.24 (0.11–0.39)	0.24 (0.11–0.39)	0.53 (0.24–0.90)	0.53 (0.24–0.90)
Capital costs^b^	Building cost	0.08 (0.03–0.13)	0.08 (0.03–0.13)	0.37 (0.12–0.89)	0.37 (0.12–0.89)
	Equipment & Furniture	0.10 (0.05–0.25)	0.13 (0.05–0.28)	0.97 (0.01–1.75)	1.00 (0.06–2.15)
	Vehicle cost	0.10 (0.00–0.75)	0.10 (0.00–0.75)	0.25 (0.00–1.95)	0.25 (0.00–1.95)
Consultation & care cost per case		4.01 (2.87–5.22)	4.91 (3.69–5.63)	10.34 (4.74–15.14)	12.37 (6.19–18.27)
**Consumable costs**					
Diagnostics		1.03 (0.80–1.27)	1.06 (0.80–1.27)	1.03 (0.80–1.27)	1.06 (0.80–1.27)
Treatment	Medicines	0.81 (0.65–0.99)	0.81 (0.65–0.99)	5.45 (N/A)	5.45 (N/A)
	Other treatment supplies	NA	NA	2.95 (N/A)	2.95 (N/A)
Consumable cost per case		1.83 (1.49–2.20)	1.87 (1.49–2.20)	9.43 (9.20–9.68)	9.46 (9.20–9.68)
Total cost per case treated		5.84 (4.86–6.85)	6.78 (5.80–7.83)	19.77 (14.14–24.60)	21.84 (15.59–27.95)

a Overheads include maintenance, training, utilities and other administration costs.

b All capital costs are annualized.

c Range across the eight health facilities.

All costs reported in constant 2022 USD.

Financial costs include resources that are paid for; economic costs reflect the full value of resources used including those which do not incur a financial cost (donated funds, goods, services or time).

Across the health centres, the economic cost per outpatient and inpatient malaria cases were strongly correlated (*r* = 0.91; *P* = 0.002). We did not find significant correlation between the number of diagnosed malaria outpatient visits (*r* = −0.11; *P* = 0.80) or inpatient nights and costs per case (*r* = −0.05; *P* = 0.91). Small and insignificant differences in costs per outpatient (difference: $0.63; p = 0.85) or inpatient case (difference: $1.47; p = 0.72) were observed between HCIIIs and HCIVs. We did not find significant correlation between time per malaria consultation and economic costs per outpatient case (*r* = 0.58; *P* = 0.13) .

### Household perspective

Overall, 614 residents from 3518 households surveyed were reported to have experienced a fever in the last 2 weeks, including 235 children aged <5 years, 230 children aged 5–15 years and 149 residents aged ≥16 years. The 614 reported suspected malaria cases came from 496 households, of which 39% had a female head of household. The 415 respondents (68%) who had recovered at the time of survey reported a mean of 3.6 days of illness (median:3; range: 0–90 days). In total, 379 (62%) respondents sought treatment for their fever, primarily outpatient treatment (350, 92%) and from a single source (342, 90%). Care was most frequently first sought from government-run health centres (152, 40%) or private clinics (99, 26%); consultation at drug shops was also common (115, 30%). Of those who sought care, 244 (64%) were tested for malaria and 208 (85%) had a positive test result. Most respondents who sought care received medications (86%), including paracetamol (*n* = 224), artemether-lumefantrine (*n* = 224), dihydroartemisinin-piperaquine (*n* = 8), IV artesunate (*n* = 15), IV quinine (*n* = 11) and amoxycillin (n = 31). Respondents who sought inpatient treatment reported a longer duration of illness than those who sought outpatient treatment (4.2 vs 3.5 days).

The mean economic cost to households was $9.71 (95% CI: 8.26–11.16) for a suspected malaria case, $12.65 (95% CI: 10.36–14.94) for a suspected malaria case that received outpatient care, and $20.29 (95% CI: 14.29–26.28) for a suspected malaria case that received inpatient care. Costs were higher for those who sought outpatient care at a private facility (as the first or second point of care) than for those who only sought treatment at a government-run health centre ($14.07 vs $10.10; difference $3.97; *P* = 0.05), with patients incurring 10 times higher costs of medicines ($1.06 vs. $0.11) and diagnostics ($0.32 vs. $0.03) at private facilities. Costs at private and public facilities were similar for inpatient cases ($21.00 vs $19.41). The mean cost for care was higher for suspected malaria cases in households headed by women as compared to men ($11.89 vs $8.23; difference $3.66; *P* = 0.01). The cost of medicines drove OOP costs for suspected outpatient ($0.72, 52%) and inpatient malaria cases ($2.18, 55%). Productivity costs accounted for 88% of household costs for suspected outpatient cases (mean: $11.07) and 76% for inpatient cases (mean: $15.44).

### Societal perspective

We estimated a societal economic cost of $15.12 (95% CI: 12.83–17.41) per suspected outpatient case of malaria and $27.21 (95% CI: 20.43–33.99) per inpatient case (Table 3). The societal costs per confirmed malaria case were higher, $19.02 (95% CI: 15.06–22.98) per outpatient and $29.29 (95% CI: 20.57–38.00) per inpatient case. Households incurred 81% of outpatient and 72% of inpatient suspected malaria costs. One-way sensitivity analyses identified productivity loss assumptions (valuation of one day, number of days reported lost, percentage of caregiver time lost, percentage of productivity loss if sick and working), and method for eliciting OOP costs as variables for which plausible variation leads to societal cost estimates for outpatient and inpatient cases that are at least 5% higher or lower than our central estimate.

**Table 3.** Disaggregated societal mean economic cost per suspected case of malaria

			Treated suspected cases		Parasitologically confirmed cases	
	All suspected cases (*n* = 614)	Untreated suspected cases (*n* = 235)	Outpatient (*n* = 350)	Inpatient (*n* = 29)	Outpatient (*n* = 190)	Inpatient (*n* = 21)
Health service costs						
Consultation	1.49	0.00	2.08	6.46	2.82	5.59
Diagnostics	0.15	0.00	0.24	0.21	0.38	0.21
Drugs	0.10	0.00	0.15	0.25	0.22	0.30
Total health service costs (95% CI)	1.73 (1.49–1.97)	0.00 N/A	2.47 (2.17–2.77)	6.92 (4.49–9.36)	3.42 (3.01–3.84)	6.10 (3.10–9.10)
Household costs						
OOP costs (Method 1)^a^	1.13	0.00	1.58	4.84	2.14	6.22
Lost time due to transport	0.37	0.00	0.58	0.78	0.64	0.91
Lost time due to waiting	0.48	0.00	0.80	0.62	1.11	0.71
Lost productivity due to illness	3.86	2.60	4.59	5.08	5.35	5.64
Lost productivity due to caregiving	3.87	1.41	5.10	8.96	6.35	9.71
Total household costs (95% CI)	9.71 (8.26–11.16)	4.02 (2.89–5.15)	12.65 (10.36–14.94)	20.29 (14.29–26.28)	15.59 (11.58–19.60)	23.19 (15.61–30.78)
Total Societal Costs (95% CI)	11.44 (9.95–12.94)	4.02 (2.89–5.15)	15.12 (12.83–17.41)	27.21 (20.43–33.99)	19.02 (15.06–22.98)	29.29 (20.57–38.00)

a OOP costs estimated from Method 1 (single question).

All costs reported in constant 2022 USD.

Economic costs presented here; financial costs found in the supplementary materials.

### Equity

In univariate analysis, we found household members aged ≥16 years were less likely to report fever (2% [149/7578] vs 5% [465/8606]; *P* < 0.001) and more likely to incur higher costs per suspected case of malaria ($18.96 vs $6.74; *P* < 0.001) than household members aged <15 years. Compared with other household members, heads of household were slightly less likely to report fever (2%[80/3518] vs 4%[534/12 668]; *P* < 0.001) but had significantly higher costs per suspected case ($23.26 vs $7.67; *P* < 0.001). There were no gender differences in fever, treatment-seeking, or costs of treatment.

**Figure 3.**
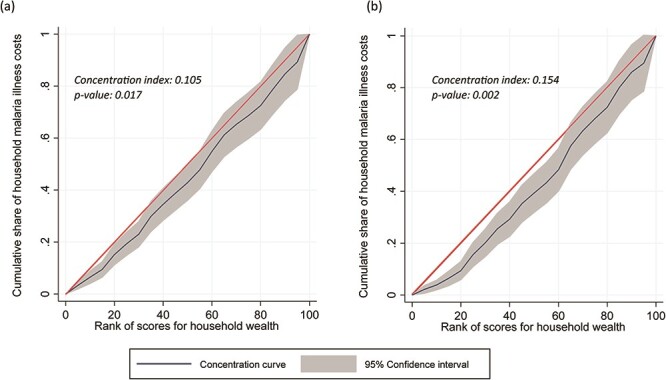
Equality in concentration of household cost per malaria episode by household socio-economic status for (a) all household members who had fever in past 2 weeks (*n* = 614) and (b) all household members surveyed (*n* = 16 189)

The concentration curves for suspected malaria cases (*n* = 614; index = 0.105; *P* = 0.02) and all household residents (*n* = 16 184; index = 0.154; *P* = 0.002) were slightly but significantly below the line of equality (Figure 3), which indicates that richer households only incurred a slightly higher share of household malaria costs than poorer households. The share of household costs were slightly more concentrated in the richer households for the full survey population because wealthier households were slightly more likely to report a fever in the past 2 weeks (wealthiest: 4% [124/3225] vs poorest: 3% [88/3046]; *r* = 0.020; *P* = 0.02) and were more likely to seek care for that fever (wealthiest: 67% [83/124] vs poorest: 50% [44/88]; *r* = 0.839; *P* = 0.02). Using national wealth quintiles, we found that mean household costs for a suspected outpatient malaria case ($9.92) accounted for a mean of 26% of monthly per capita consumption ($37.90) in the poorest quintile, 18% in Q2 ($11.35/$62.32), 17% in Q3 ($14.76/$87.49), 10% in Q4 ($12.28/$126.10) and 8% in the wealthiest quintile ($21.82/$285.86).

Differences in treatment-seeking behaviour for suspected malaria were observed across sub-regions, although numbers in some areas were small. Treatment for suspected malaria was less commonly sought in the Busoga (42%[63/151]) and Acholi sub-regions (48%[44/92]) than in Bunyoro (67%[48/72]), Teso (67%[71/106]) and Lango sub-regions (83%[120/145]). Household costs per suspected case of malaria were not significantly different across regions (*P* = 0.24), suggesting that costs were similar in different transmission intensities. Bukedi sub-region had the lowest mean household costs per case of malaria ($2.57; 95% CI: −0.77 to –5.92) and Acholi sub-region the highest ($12.19; 95% CI: 10.23–15.15).

In the multivariable analyses utilizing a two-part model, wealth percentile, sub-region and age were retained in the final parsimonious model (Table 4). As all households were located in rural areas, and data on education level were unavailable, these variables were not considered. Head of household status was excluded due to collinearity with age, and gender was not included as it did not enhance the model’s fit.The logit model indicated that age perfectly predicted whether costs were incurred; wealth (OR: 1.01; 95% CI: 1.00–1.02) also drove this variation, but only slightly. Among those households with non-zero illness costs, the GLM model indicated that adjusting for the other covariates in the model, there was a significant association between age (OR: 2.11; 95% CI: 1.67–266) and wealth (OR 1.01: 95% CI: 1.00–1.01) and costs incurred. The overall marginal effects combining both parts of the two-part model indicated household members aged ≥16 years incurred $4.99 more costs per case compared to those <15 years (*P* < 0.001) and there is a $0.06 increase in cost per percentile increase in household wealth (*P* = 0.001). We compared our parsimonious model with a theory-driven model that included gender and found the results to be robust across multiple specifications, as there were no substantial differences in the coefficients, *P*-values, or model metrics (Supplementary materials).

**Table 4.** Drivers of household cost per suspected case of malaria

			Two-part model							
			Logit model *n* = 463			General linearized model *n* = 471			Marginal effects	
Explanatory variables		Mean	Odds ratio	*P*-value	95% CI	Odds ratio	*P*-value	95% CI	Coefficient	*P*-value
Age	<15 years	6.74	Omitted ^a^	-	-	*Ref*	*Ref*	*Ref*	*Ref*	*Ref*
	16+ years	18.96	Omitted ^a^	-	-	2.11	<0.001	1.67–2.66	4.99	<0.001
Wealth	Percentile	NA	1.01	0.01	1.00–1.02	1.01	0.006	1.00–1.01	0.06	0.001
Sub-region	North Buganda	12.19	*Ref*	*Ref*	*Ref*	*Ref*	*Ref*	*Ref*	*Ref*	*Ref*
	Bunyoro	9.20	0.39	0.92	0.15–5.41	1.08	0.88	0.34–3.07	0.18	0.94
	West Nile	8.14	1.82	0.59	0.27–12.2	1.41	0.57	0.43–4.65	2.45	0.38
	Acholi	9.55	0.70	0.69	0.12–4.01	2.26	0.17	0.71–7.14	3.78	0.17
	Lango	9.74	2.70	0.26	0.47–15.2	1.83	0.30	0.59–5.69	4.89	0.56
	Teso	11.14	1.86	0.49	0.32–10.9	1.78	0.32	0.57–5.56	4.13	0.11
	Busoga	9.71	0.51	0.43	0.09–2.77	1.69	0.36	0.54–5.21	1.14	0.65
	Bukedi	2.57	Omitted ^b^	-	-	0.39	0.19	0.09–1.61	−2.33	0.32
	Tooro	4.93	Omitted ^b^	-	-	0.33	0.37	0.02–3.82	−2.56	0.33
	Karamoja ^c^	-	-	-	-	-	-		-	-
	Pseudo *R*^2^		0.0717							
	Deviance					472.9				
	Pearson					577.8				
	AIC					23.37				
	BIC					−2358				

a Variable omitted from model because category predicted success perfectly.

b Omitted from model due to collinearity.

c No observations.

All costs reported in constant 2022 USD.

### Economic burden

Approximately 22 000 malaria-related deaths and 13 million cases of malaria occurred in Uganda, 2% of which were severe and treated as inpatient cases (*n* = 293 026) (World Health Organization, 2022). We estimated a net present value of lost productivity of $18 199 per life lost. In 2021, our estimates indicate that malaria illness cost Ugandan society $577 million ($12.57 per capita), of which 68% were productivity losses from mortality and 32% were associated with illness episodes. Of the latter, 92% were from uncomplicated malaria and 84% were borne by households. Best- and worst-case scenarios produced societal costs estimates ranging from $302 million to $1.09 billion USD.

## Discussion

Our estimates of the societal cost per suspected malaria case treated on an outpatient ($15.12 USD) or inpatient ($27.21 USD) basis quantify the economic value of preventing malaria in Uganda. Extrapolating our findings to the whole country and including mortality-related productivity losses, we estimated that in 2021, malaria cost Uganda $577 million USD, roughly 1.4% of Uganda’s GDP, which is slightly higher than previously reported in Tanzania (1.1%) and Uganda (0.7%) (Jowett and Miller, 2005; Orem *et al*., 2012; World Bank, 2023b). These costs were not distributed equitably across Ugandan society; for both outpatient and inpatient treatment, >70% of costs were borne by households. Our estimate of mean societal costs per suspected treated outpatient case ($15.12) represents 11% of mean rural monthly consumption expenditure ($144) and OOP costs per case ($1.58) represents 5% of median monthly income per capita ($44) (Ugandan Bureau of Statistics, 2021). Our finding that the cost per case of malaria did not vary by wealth and comprised a three times greater share of the consumption expenditure of the poorest quintile (26%) compared to the wealthiest (8%) indicates that the distribution of costs is not equitable. This places poorer households at higher risk of ‘catastrophic’ health expenditure, which occurs when a substantial percentage (10–25%) of total monthly income is spent on OOP medical costs (Alam and Mahal, 2014; O’Donnell, 2019). Productivity losses, which are highly sensitive to how time is valued, drove household costs and overall societal costs, suggesting methodological transparency is crucial to interpreting these and other study results.

Our estimates of the societal cost per case of illness in Uganda were substantially lower than the most recent previous estimate (53 USD 2011 per case) (Orem *et al*., 2011). However, Orem *et al.* did not distinguish between outpatient and inpatient malaria, only included government expenditure on antimalarials when estimating health service costs and reported more days away from work due to illness (7.8 vs 2.3), leading to higher productivity loses ($49.30 vs $8.59 per episode) than our study. WHO-CHOICE cost estimates for Ugandan outpatient visits ($3.28–$4.79 USD 2022) are slightly lower than ours ($4.91 USD 2022; range: $3.69–5.63), but their estimates for an inpatient bed-day ($14.13–$15.91 USD 2022) are more than double our consultation and care economic costs divided by our average inpatient stay ($5.37 USD 2022; range: $3.10–$9.13), suggesting the WHO-CHOICE inpatient values could be overestimates. We also found that outpatient care drove 92% of the economic burden of malaria treatment in Uganda in 2021, which is above the range reported in a systematic review (44–74%) (Sicuri *et al*., 2011). Past estimates likely captured treatment with less effective drugs, leading to longer recovery time and more productivity losses. Reduced antimalarial costs, a high case burden resulting in economies of scale at laboratories and limited inpatient services could explain the lower costs per case in Uganda compared to other settings. Additionally, in high burden settings like Uganda, where the population has greater antimalarial immunity acquired through repeated exposure to malaria parasites, fewer cases will progress to severe malaria (Rogier *et al*., 1999; White, 2018; World Health Organization, 2022). Finally, the health facilities included in our study may have not captured the most severe and expensive cases, which may have been treated at higher-level facilities or referral hospitals. Our study confirmed that a smaller number of questions (Method 1) yield a higher OOP cost estimate, in line with previous methodological literature (Heijink *et al*., 2011; Agorinya *et al*., 2021). Other studies have found lower illness costs for children compared to adults (Alonso *et al*., 2019), lower costs for patients from poorer compared to wealthier household (Onwujekwe *et al*., 2010; Gunda *et al*., 2017; Singh *et al*., 2019) and equal costs across household socio-economic groups (Somi *et al*., 2007; Castillo-Riquelme *et al*., 2008). Although our cost per case estimates is generally lower than other studies, we think the evolution of malaria treatment and the transparent methodological choices we made in terms of productivity losses explain these differences.

Our findings are generalizable to rural Uganda, where 73% of the population lives (Ugandan Bureau of Statistics, 2021). Urban government health facilities likely have similar consumable costs, as all are provided by the National Medical Stores. However, we predict higher staff salaries, OOP payments and productivity losses (due to higher household consumption per day) in urban areas. Level II health centres, which were not included in our study, are the most numerous government health centres in Uganda. Although we did not see a significant difference between costs at HCIII and HCIV, it is possible that the costs incurred at lower-level health centres could be different. By focusing only on HCIII and HCIV, we may have over-estimated the costs of outpatient care and underestimated inpatient costs. Although our study took place during the COVID-19 pandemic and there is evidence that stock-outs of RDTs and drugs negatively affected malaria treatment nationwide (Mumali *et al*., 2023, Zalwango *et al*., 2023), evidence from our study sites during the same time period suggests that the pandemic had no major effects on indicators of malaria disease burden and case management and only a slight effect on delivery of RDTs and AL (Namuganga *et al*., 2021). We disaggregated costs of outpatient and inpatient visits to present our results in the most precise manner possible; these estimates can be used for uncomplicated and severe case estimates in future economic evaluations. Although the economic burden estimate is specific to Uganda, we believe that our cost-per-case estimates and equity findings can be useful in other malaria endemic populations where local estimates are not available, specifically where malaria transmission is high and health facilities are similarly resourced.

This study has additional limitations. First, we focused on health centres that have been designated sites for enhanced malaria surveillance and receive additional support such as ensured supply of RDTs, which may have increased standard of care. Second, our study survey population was limited to target areas surrounding these health centres. We found a higher proportion of febrile children who sought treatment (27%; 95% CI: 24–30%) than the 2018–2019 Malaria Indicator Survey (MIS) (13%; 95% CI: 11–15%) (World Health Organization, 2022), suggesting those close to a government-run health centre may be more likely to seek treatment and use public over private facilities due to closer proximity. Patients may be more likely to be tested and/or treated for malaria in government-run health centres than in private facilities. In our survey, the proportion of children under five reported to have fever in the preceding 2 weeks was lower than the 2018–2019 MIS (8% [95% CI: 7–9%] vs 68% [95% CI: 62–74%]). The reasons for this are unclear, but may reflect reporting or recall bias, or lower fever prevalence. Third, we based our calculations on the medicines prescribed assuming that medicines were always available at the health centre and may have overestimated outpatient medicine costs at the health centres. Finally, since we included all fevers, we may have included non-malaria illness costs in our results. However, if we limited our study population to those with parasitological confirmation, we would have skewed the population towards people who sought care in a government-run health centre and would have underestimated the burden of malaria.

Our results have several important implications for policy and future research. We found that the overall economic burden at a national level was exceptionally high, which indicates urgent need to scale-up malaria prevention efforts. Despite Uganda’s no-user fee policy at government health facilities, we found that households continue to incur OOP costs for consultation, diagnostics and medicines, suggesting stockouts or demands for informal payment at the health centres (Kwesiga *et al*., 2020). Addressing these inequities would require improving access to quality health services to reduce household costs. Finally, understanding drivers of variation in household costs per case across sub-regions (range: $4.01–12.19) could help policymakers target interventions. To monitor trends in household treatment costs over time and space, we suggest researchers explore the value of including brief question(s) regarding OOP costs of treatment on standard malaria surveys. Up-to-date and generalizable cost-of-illness studies could inform malaria control decision-making and studies that provide estimates for different geographic and demographic groups can aid research on the cost-effectiveness and equity of future malaria control strategies.

## Supplementary Material

czae093_Supp

## Data Availability

The data underlying this article will be shared on reasonable request to the corresponding author.

## Reflexivity statement

The authors group for this paper is equally balanced in terms of gender, with women listed as the first, second and last author. At least half of the authors on this paper are considered junior investigators or early-career researchers. The first author is a female of American nationality who has lived and worked in Uganda for the past decade, 12 of the 16 authors are of East African nationality.
