# Supplementary material for: Who pays to treat malaria and how much? Analysis of the cost of illness, equity and economic burden of malaria in Uganda
Source: Health Policy Plan. 2024 Oct 15;40(1):52–65. doi: 10.1093/heapol/czae093 (PMC11724642; doi:10.1093/heapol/czae093)
Supplement: czae093_Supp [file czae093_supp.zip › czae093_Supp/COI Supplementary Material_V3.docx]

Supplementary Material

Who pays to treat malaria, and how much? Analysis of the cost of illness, equity, and economic burden of malaria in Uganda

Contents

[Methods 3](#_Toc177644954)

[Supplementary Table S1 Cost-of-illness evaluation checklist 3](#_Toc177644955)

[Supplementary Table S2: Description of perspective and costs 5](#_Toc177644956)

[Supplementary Table S3: Micro-costing inputs to treat an inpatient case of malaria at health centre 6](#_Toc177644957)

[Supplementary Table S4: Parameters for economic burden -analysis 7](#_Toc177644958)

[Results 9](#_Toc177644959)

[Health Service Costs 9](#_Toc177644960)

[Supplementary Table S5: Health service costs per clinically diagnosed malaria case (a) financial costs and (b) economic costs 9](#_Toc177644961)

[Supplementary Figure S1: Health service economic cost per case of clinically diagnosed malaria, by health centre 11](#_Toc177644962)

[Household Descriptive Statistics and Costs 12](#_Toc177644963)

[Supplementary Figure S2: Community survey data collection cascade 12](#_Toc177644964)

[Supplementary Table S6: Descriptive information for household members with suspected malaria 13](#_Toc177644965)

[Supplementary Table S7: Treatment seeking characteristics for household members with suspected malaria 14](#_Toc177644966)

[Supplementary Table S8: Household mean economic costs per malaria episode by cost category, costing method, type of treatment 16](#_Toc177644967)

[Supplementary Table S9: Treatment seeking behaviour and household economic costs per suspected case of malaria by equity-relevant variables 17](#_Toc177644968)

[Supplementary Figure S3 Methods for estimating household out-of-pocket costs 18](#_Toc177644969)

[Supplementary Table S10: Household mean costs per suspected case by National SES Quintile and percentage of per capita consumption 19](#_Toc177644970)

[Supplementary Table 11: Drivers of household cost per suspected case of malaria, alternative theory-driven model 20](#_Toc177644971)

[Societal Costs 21](#_Toc177644972)

[Supplementary Table S12: Disaggregated societal mean financial cost per suspected case of malaria 21](#_Toc177644973)

[Supplementary Table S13: Sensitivity Analysis of societal cost per case of suspected malaria (a) input parameters and justification and (b) cost outputs 22](#_Toc177644974)

[Supplementary Figure S4: Deterministic sensitivity analysis for societal costs; tornado diagram of (a) treated outpatient suspected malaria and (b) treated inpatient suspected malaria 25](#_Toc177644975)

[Supplementary Table S14: Economic burden of malaria in Uganda 27](#_Toc177644976)

# Methods

### Supplementary Table S1 Cost-of-illness evaluation checklist

Checklist items are taken verbatim from *Larg and Moss, 2011*. The left-hand column indicates where the given item was reported in the text

| **Checklist item** | **Location where item is reported** |
| --- | --- |
| **(1) Analytical framework: what costs should have been measured?** |  |
| (a) What was the motivation and perspective of the study? | Page 5 |
| (b) Was the appropriate epidemiologic approach taken? | Page 5 |
| (c) Was the study question well specified? | Page 3 and 5 |
| (i) Were all relevant, non-trivial cost components and their stakeholders identified? | Page 6,10; Supp. Table S2 |
| (ii) Were necessary timeframes specified? | Page 4 |
| (iii) Was a case of disease or risk factor adequately and appropriately defined? | Page 5 |
| (iv) Was the counterfactual population occurrence plausible and meaningful? | Page 5 |
| **(2) Methodology and data: how well were resource use and productivity losses measured?** | |
| (a) Was an appropriate method(s) of quantification used, such that |  |
| (i) additional, or excess, costs were measured? | Page 8 |
| (ii) only costs specific to (caused by) the health problem were included (confounders  controlled?) | Page 6 |
| (iii) all important effects were captured? | Page 8 |
| (iv) important differences across subpopulations were accounted for? | Page 8 |
| (v) the required level of detail could be provided? | Page 8-10 |
| (b) Was the resource quantification method(s) well executed? |  |
| (i) For population-based studies, were cost allocation methods, data and  assumptions valid? | Page 9; Figure 2 |
| (ii) For person-based studies, were appropriate statistical tests performed and  reported? | Page 9, 11, 12 |
| (iii) Were data representative of the study population? | Page 19-20 |
| (iv) Were there any other relevant resource quantification issues? | Page 17; Supp.Table S13 |
| (c) Were healthcare resources valued appropriately? | Page 9, 10 |
| (d) Was the approach for valuing production losses justified, and assumptions valid? | Page 9, 10, 12, 18 |
| (e) Was the inclusion of intangible costs appropriate: |  |
| (i) Was double counting of mortality-related production losses avoided? | Page 12; Supp. Table S14 |
| (ii) Were losses valued appropriately, given the study’s perspective? | Not applicable |
| **(3) Analysis and reporting** |  |
| (a) Did the analysis address the study question? | Page 3, 15, 15, 16, 17 |
| (b) Was a range of estimates presented? | Page 13-17; Supp. Table S7, S8 |
| (c) Were the main uncertainties identified? | Page 10, 15: Supp. Table S12, S13 |
| (d) Was a sensitivity analysis performed on: |  |
| (i) important (uncertain) parameter estimates? | Page 10, 15: Supp. Table S12, S13 |
| (ii) key assumptions? (including the counterfactual) | Page 10, 15: Supp. Table S12, S13 |
| (iii) point estimates? (based on confidence or credible intervals) | Page 10, 15: Supp. Table S12, S13 |
| (e) Was adequate documentation and justification given for cost components, data and  sources, assumptions and methods? | Page 8, 9, 10, 12; Supp. Table S3, S12, S13 |
| (f) Was uncertainty around the estimates and its implications adequately discussed? | Page 15; Supp. Table S12 |
| (g) Were important limitations discussed regarding the cost components, data,  assumptions and methods? | Page 19, 20 |
| (h) Were the results presented at the appropriate level of detail to answer the study  question (cost components; disease subtypes, severity, stage; subpopulation groups,  cost bearers)? | Table 2, 3; Supp. Table S4, S7, S8 |

### Supplementary Table S2: Description of perspective and costs

|  | | Economic Costs | |
| --- | --- | --- | --- |
|  |  | Financial Cost | Additional opportunity costs |
| Societal Perspective | Health Service  *Source: Health centre costing* | Consultation & care cost   - Employee salaries - Capital costs (buildings, equipment, furniture, vehicles) - Overheads (maintenance, training)   Consumables   - Diagnostics - Medicines - Other treatment supplies | Consultation & care cost   - Volunteer staff (salaries estimated by qualification) - Capital costs (donated equipment, furniture, vehicles)   Consumables   - Diagnostic supplies donated by IDRC |
|  | Household  *Source: Cross-sectional household survey* | Out-of-pocket payments for   - Consultation - Diagnostic - Medicines - Transport - Special food | Productivity loss due to   - Time lost to illness - Time lost to caregiving - Time lost due to transportation - Time lost waiting |

### Supplementary Table S3: Micro-costing inputs to treat an inpatient case of malaria at health centre

| **Labour** |  | **Unit cost** | **Number of units** | **Total cost** | **Total cost plus 10% wastage** |
| --- | --- | --- | --- | --- | --- |
|  | Nurse time – intake ( 15 minutes) | 0.28 | 1 | 0.28 |  |
|  | Nurse time – check every 4 hours (10 minutes) | 0.18 | 12 | 2.21 |  |
|  | Lab Tech time (20 minutes) | 0.37 | 1 | 0.37 |  |
|  | Pharmacist time (15 minutes) | 0.28 | 2 | 0.55 |  |
|  | Discharge (30 minutes) | 0.55 | 1 | 0.55 |  |
|  | Clinician rounds (5 minutes x 2 times a day) | 0.09 | 4 | 0.37 |  |
|  | **Total Labor** |  |  | **4.32** |  |
| **Consumables** | |  |  |  |  |
|  | **Diagnostics** |  |  |  |  |
|  | RDT test | 0.68 | 1.4 | 0.95 |  |
|  | Microscopy smear reading | 0.29 | 0.3 | 0.09 |  |
|  | Total | - |  | 1.03 |  |
|  | **Medicines** |  |  |  |  |
|  | Artesunate (3 ampules minimum) | 1.79 | 3 | 5.37 |  |
|  | Paracetamol | 0.01 | 24 | 0.13 |  |
|  | Rehydration bag | 0.41 | 1 | 0.41 |  |
|  | Artemether-lumefantrine | 0.66 | 1 | 0.66 |  |
|  | Total |  |  | 6.58 |  |
|  | **Other** |  |  |  |  |
|  | Gloves (pair) | 0.19 | 5 | 0.94 | 1.03 |
|  | Alcohol rub per interaction (15 ml) | 0.12 | 5 | 0.60 | 0.66 |
|  | Alcohol swab | 0.01 | 5 | 0.06 | 0.07 |
|  | Canula | 0.12 | 1 | 0.12 | 0.13 |
|  | Syringes | 0.05 | 3 | 0.14 | 0.16 |
|  | Cotton | 0.02 | 5 | 0.12 | 0.13 |
|  | Gauze | 0.01 | 5 | 0.04 | 0.04 |
|  | Set infusion | 0.65 | 1 | 0.65 | 0.71 |
|  | Tape | 0.02 | 1 | 0.02 | 0.02 |
|  | Total |  |  | 2.68 | 2.95 |
|  | **Total Consumables** |  |  | **10.29** | **10.56** |

*All costs reported in constant USD 2022.*

### Supplementary Table S4: Parameters for economic burden -analysis

| **Input** | **Base case** | **Best Case** | **Worst Case** | **Justification** |
| --- | --- | --- | --- | --- |
| **Costs per malaria case** |  |  |  | Present study – costs per suspected case of malaria in constant USD 2022 |
| Untreated household | 4.02 | 2.89 | 5.51 |  |
| Outpatient health service, economic | 2.47 | 2.17 | 2.77 |  |
| Outpatient household, economic | 12.65 | 10.36 | 14.94 |  |
| Inpatient health service, economic | 6.92 | 4.49 | 7.92 |  |
| Inpatient household, economic | 20.29 | 14.29 | 26.28 |  |
| Outpatient health service, financial | 2.09 | 1.84 | 2.34 |  |
| Outpatient household, financial | 1.58 | 1.27 | 1.89 |  |
| Inpatient health service, financial | 5.86 | 3.80 | 7.92 |  |
| Inpatient household, financial | 4.84 | 2.20 | 7.49 |  |
| **Morbidity** |  |  |  |  |
| Total population | 45 853 776 |  |  | 2022 World Malaria Report^1^ |
| Number of malaria cases | 13 023 397 | 8 637 000 | 20 610 000 | 2022 World Malaria Report^1^ |
| % of children under 5 who sought treatment for fever | 86.9 | 84.7 | 88.8 | 2022 World Malaria Report^1^ |
| % of cases severe malaria | 3 | 1 | 5 | Uganda Ministry of Health expert opinion and 2022 World Malaria Report^1^ |
| % of severe malaria hospitalized | 75 | 50 | 80 | 2022 World Malaria Report^1^ |
| **Mortality** |  |  |  |  |
| Number of malaria deaths | 21 699 | 16 500 | 30 900 | 2022 World Malaria Report ^1^ |
| Mean age of death (years) | 5 |  |  | Assumption |
| Minimum age to start working (years) | 12 | 15 | 10 | Assumption |
| Life expectancy at 5 years (years) | 65 |  |  | World Health Organization^2^ |
| GDP per capita | 884 |  |  | World Bank ^3^ |
| % of GDP on Health Expenditure | 4% |  |  | World Bank ^3^ |
| Non health expenditure GDP | 849 |  |  | GDP per capita x (1-% GDP on health expenditure) |
| Discount rate | 0.03 | 0.01 | 0.05 | iDSI reference case ^4^ |
| Net present value of lost productivity - per lost life discounted | 18 199 | 9511 | 34 031 | Non-health expenditure GDP x working years |

References

1 World Health Organization. World malaria report 2022. 2022.

2 World Health Organization. Life Tables by Country. 2020. https://www.who.int/data/gho/data/indicators/indicator-details/GHO/gho-ghe-life-tables-by-country (accessed June 14, 2023).

3 World Bank. Current health expenditure (% of GDP) - Uganda. https://data.worldbank.org/indicator/SH.XPD.CHEX.GD.ZS?locations=UG (accessed March 22, 2023).

4 Wilkinson T, Sculpher MJ, Claxton K, *et al.* The International Decision Support Initiative Reference Case for Economic Evaluation: An Aid to Thought. *Value in Health* 2016; **19**: 921–8.

#

# Results

## Health Service Costs

### Supplementary Table S5: Health service costs per clinically diagnosed malaria case (a) financial costs and (b) economic costs

**(a)**

| **Financial Cost Category** | | | | **HC1** | **HC2** | **HC3** | **HC4** | **HC5** | **HC6** | **HC7** | **HC8** | **All HEALTH CENTERS (Range)** |
| --- | --- | --- | --- | --- | --- | --- | --- | --- | --- | --- | --- | --- |
| **Outpatient Malaria** | Consultation & care costs | Recurrent Costs | Labor | 3.31 | 3.38 | 3.62 | 3.89 | 3.99 | 3.86 | 3.39 | 2.49 | 3.49 (2.49 – 3.99) |
|  |  |  | Overheads* | 0.11 | 0.36 | 0.19 | 0.13 | 0.39 | 0.23 | 0.27 | 0.24 | 0.24 (0.11 – 0.39) |
|  |  | Capital Costs § | Building Cost | 0.06 | 0.03 | 0.06 | 0.10 | 0.13 | 0.13 | 0.04 | 0.08 | 0.08 (0.03 – 0.13) |
|  |  |  | Equipment & Furniture | 0.05 | 0.10 | 0.11 | 0.11 | 0.07 | 0.25 | 0.05 | 0.05 | 0.10 (0.05 – 0.25) |
|  |  |  | Vehicle Cost | 0.00 | 0.01 | 0.00 | 0.01 | 0.00 | 0.75 | 0.00 | 0.00 | 0.10 (0.00 – 0.75) |
|  |  | Consultation & care cost per case | | 3.54 | 3.88 | 3.99 | 4.24 | 4.58 | 5.22 | 3.75 | 2.87 | 4.01 (2.87 – 5.22) |
|  | Consumable costs | Diagnostics | | 1.07 | 1.21 | 0.80 | 1.11 | 0.84 | 0.90 | 1.27 | 1.00 | 1.03 (0.80 – 1.27) |
|  |  | Treatment | Medicines | 0.93 | 0.68 | 0.78 | 0.77 | 0.65 | 0.74 | 0.93 | 0.99 | 0.81 (0.65 – 0.99) |
|  |  |  | Other treatment supplies | N/A | N/A | N/A | N/A | N/A | N/A | N/A | N/A | N/A |
|  |  | Consumable cost per case | | 2.00 | 1.89 | 1.58 | 1.88 | 1.49 | 1.64 | 2.20 | 1.98 | 1.83 (1.49 – 2.20) |
|  | Total Cost per case treated | | | 5.54 | 5.77 | 5.57 | 6.12 | 6.06 | 6.85 | 5.95 | 4.86 | 5.84 (4.86 – 6.85) |
| **Inpatient Malaria** | Consultation & care costs | Recurrent Costs | Labor | 8.15 | 8.46 | 6.77 | 13.30 | 5.84 | 10.04 | 10.73 | 2.49 | 8.22 (2.49 – 13.30) |
|  |  |  | Overheads* | 0.27 | 0.90 | 0.36 | 0.45 | 0.57 | 0.59 | 0.84 | 0.24 | 0.53 (0.24 – 0.90) |
|  |  | Capital Costs § | Building Cost | 0.17 | 0.12 | 0.14 | 0.27 | 0.89 | 0.84 | 0.27 | 0.26 | 0.37 (0.12 – 0.89) |
|  |  |  | Equipment & Furniture | 0.09 | 1.03 | 0.01 | 1.03 | 1.71 | 1.71 | 0.39 | 1.75 | 0.97 (0.01 – 1.75) |
|  |  |  | Vehicle Cost | 0.00 | 0.02 | 0.01 | 0.03 | 0.01 | 1.95 | 0.01 | 0.00 | 0.25 (0.00 – 1.95) |
|  |  | Consultation & care cost per case | | 8.68 | 10.53 | 7.29 | 15.08 | 9.02 | 15.14 | 12.25 | 4.74 | 10.34 (4.74 – 15.14) |
|  | Consumable costs | Diagnostics | | 1.07 | 1.21 | 0.80 | 1.11 | 0.84 | 0.90 | 1.27 | 1.00 | 1.03 (0.80 – 1.27) |
|  |  | Treatment | Medicines | 5.45 | 5.45 | 5.45 | 5.45 | 5.45 | 5.45 | 5.45 | 5.45 | 5.45 (N/A) |
|  |  |  | Other treatment supplies | 2.95 | 2.95 | 2.95 | 2.95 | 2.95 | 2.95 | 2.95 | 2.95 | 2.95 (N/A) |
|  |  | Consumable cost per case | | 9.48 | 9.62 | 9.20 | 9.52 | 9.25 | 9.30 | 9.68 | 9.40 | 9.43 (9.20 – 9.68) |
|  | Total Cost per case treated | | | 18.16 | 20.15 | 16.49 | 24.60 | 18.27 | 24.44 | 21.93 | 14.14 | 19.77 (14.14 – 24.60) |

**(b)**

| **Economic Cost Category** | | | | **HC1** | **HC2** | **HC3** | **HC4** | **HC5** | **HC6** | **HC7** | **HC8** | **ALL HEALTH CENTERS** |
| --- | --- | --- | --- | --- | --- | --- | --- | --- | --- | --- | --- | --- |
| **Outpatient Malaria** | Consultation & care costs | Recurrent Costs | Labor | 3.46 | 3.97 | 4.67 | 4.65 | 4.95 | 4.24 | 5.27 | 3.75 | 4.37 (3.46 – 5.27) |
|  |  |  | Overheads* | 0.11 | 0.36 | 0.19 | 0.13 | 0.39 | 0.23 | 0.27 | 0.24 | 0.24 (0.11 – 0.39) |
|  |  | Capital Costs § | Building Cost | 0.06 | 0.03 | 0.06 | 0.10 | 0.13 | 0.13 | 0.04 | 0.08 | 0.08 (0.03 – 0.13) |
|  |  |  | Equipment & Furniture | 0.05 | 0.10 | 0.13 | 0.21 | 0.09 | 0.28 | 0.05 | 0.13 | 0.13 (0.05 – 0.28) |
|  |  |  | Vehicle Cost | 0.00 | 0.01 | 0.00 | 0.01 | 0.00 | 0.75 | 0.00 | 0.00 | 0.10 (0.00 – 0.75) |
|  |  | Consultation & care cost per case | | 3.69 | 4.47 | 5.05 | 5.10 | 5.56 | 5.62 | 5.63 | 4.21 | 4.91 (3.69 – 5.63) |
|  | Consumable costs | Diagnostics | | 1.18 | 1.21 | 0.80 | 1.25 | 0.84 | 0.92 | 1.27 | 1.00 | 1.06 (0.80 – 1.27) |
|  |  | Treatment | Medicines | 0.93 | 0.68 | 0.78 | 0.77 | 0.65 | 0.74 | 0.93 | 0.99 | 0.81 (0.65 – 0.99) |
|  |  |  | Other treatment supplies | - | - | - | - | - | - | - | - | NA |
|  |  | Consumable cost per case | | 2.12 | 1.89 | 1.58 | 2.02 | 1.49 | 1.65 | 2.20 | 1.98 | 1.87 (1.49 – 2.20) |
|  | Total Cost per case treated | | | 5.80 | 6.36 | 6.63 | 7.11 | 7.04 | 7.27 | 7.83 | 6.20 | 6.78 (5.80 – 7.83) |
| **Inpatient Malaria** | Consultation & care costs | Recurrent Costs | Labor | 8.50 | 9.93 | 8.72 | 15.92 | 7.24 | 11.02 | 16.67 | 3.75 | 10.22 (3.75 – 16.67) |
|  |  |  | Overheads* | 0.27 | 0.90 | 0.36 | 0.45 | 0.57 | 0.59 | 0.84 | 0.24 | 0.53 (0.24 – 0.90) |
|  |  | Capital Costs § | Building Cost | 0.17 | 0.12 | 0.14 | 0.27 | 0.89 | 0.84 | 0.27 | 0.26 | 0.37 (0.12 – 0.89) |
|  |  |  | Equipment & Furniture | 0.17 | 0.89 | 0.06 | 0.47 | 1.87 | 2.15 | 0.48 | 1.94 | 1.00 (0.06 – 2.15) |
|  |  |  | Vehicle Cost | 0.00 | 0.02 | 0.01 | 0.03 | 0.01 | 1.95 | 0.01 | 0.00 | 0.25 (0.00 – 1.95) |
|  |  | Consultation & care cost per case | | 9.11 | 11.86 | 9.29 | 17.14 | 10.57 | 16.55 | 18.27 | 6.19 | 12.37 (6.19 – 18.27) |
|  | Consumable costs | Diagnostics | | 1.18 | 1.21 | 0.80 | 1.25 | 0.84 | 0.92 | 1.27 | 1.00 | 1.06 (0.80 – 1.27) |
|  |  | Treatment | Medicines | 5.45 | 5.45 | 5.45 | 5.45 | 5.45 | 5.45 | 5.45 | 5.45 | 5.45 (N/A) |
|  |  |  | Other treatment supplies | 2.95 | 2.95 | 2.95 | 2.95 | 2.95 | 2.95 | 2.95 | 2.95 | 2.95 (N/A) |
|  |  | Consumable cost per case | | 9.59 | 9.62 | 9.20 | 9.65 | 9.25 | 9.32 | 9.68 | 9.40 | 9.46 (9.20 – 9.68) |
|  | Total Cost per case treated | | | 18.70 | 21.48 | 18.49 | 26.79 | 19.82 | 25.87 | 27.95 | 15.59 | 21.84 (15.59 – 27.95) |

******* *Overheads include maintenance, training, utilities and other administration costs.*

*§ All capital costs are annualized.*

*All costs are reported in constant USD 2022.*

*Financial costs include resources that are paid for; economic costs reflect the full value of resources used including those which do not incur a financial cost, such as donated funds, goods, services or time.*

Supplementary Figure S1: Health service economic cost per case of clinically diagnosed malaria, by health centre

*All costs reported in constant USD 2022.*

## Household Descriptive Statistics and Costs

### Supplementary Figure S2: Community survey data collection cascade

*
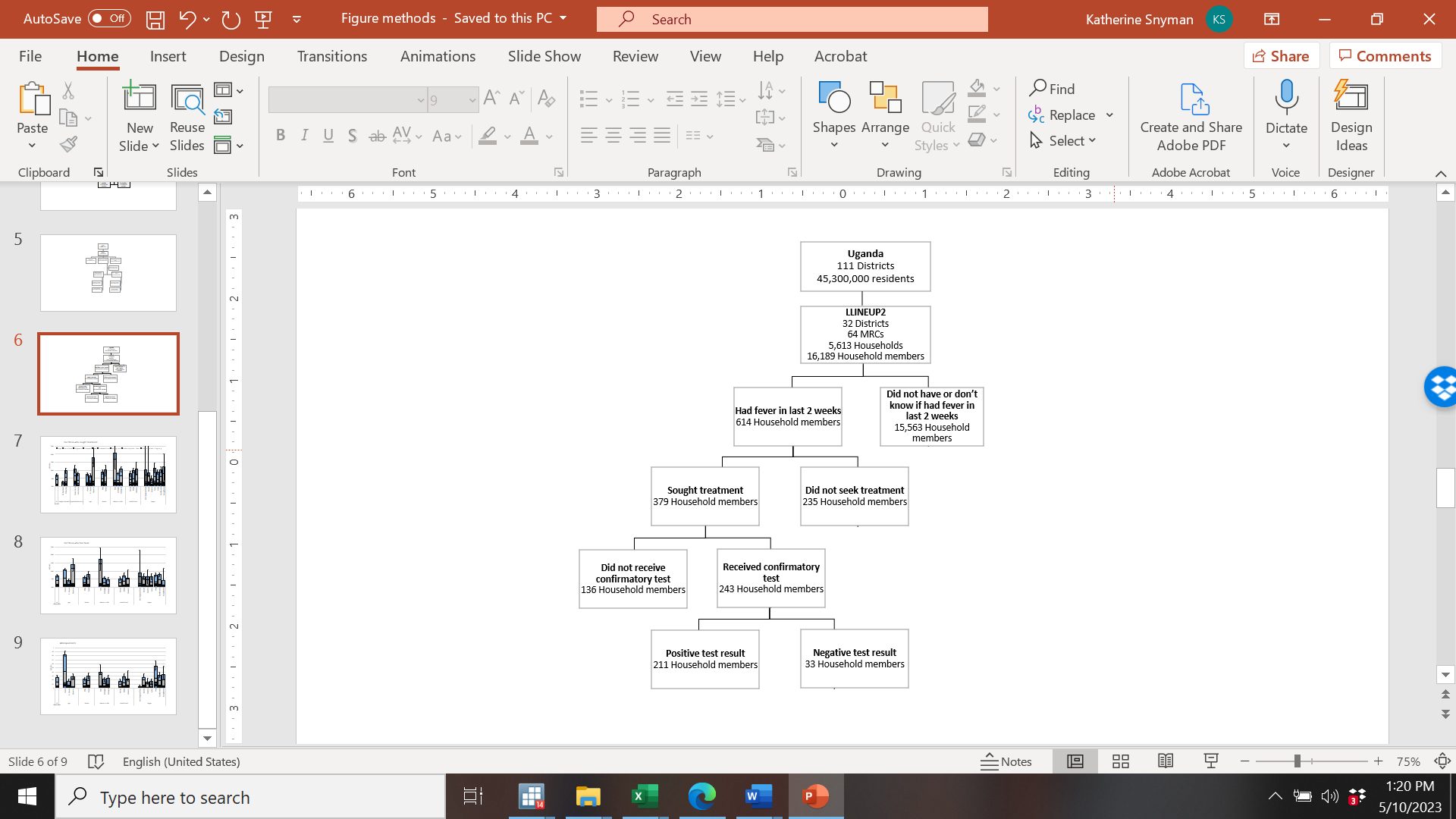
*

### Supplementary Table S6: Descriptive information for household members with suspected malaria

### Supplementary Table S7: Treatment seeking characteristics for household members with suspected malaria

| **Variable** | **Response** | **Place of care** | | |
| --- | --- | --- | --- | --- |
|  |  | **All**  **n=379** | **Only Public §**  **n=138** | **Any Private †**  **n=241** |
| First place of treatment | Government health facility | 152 (40%) | 125 (91%) | 27 (11%) |
|  | Community Health Worker | 13 (3%) | 13 (9%) | 0 (0%) |
|  | Private health facility | 99 (26%) | 0 (0%) | 99 (41%) |
|  | Dispensary / Shop/kiosk | 115 (30%) | 0 (0%) | 115 (48%) |
| Reason to choose first provider | Availability of medicines | 156 (41%) | 12 (9%) | 144 (60%) |
|  | Proximity | 96 (25%) | 55 (40%) | 41 (17%) |
|  | Inexpensive | 57 (15%) | 40 (29%) | 17 (7%) |
|  | Good reputation | 24 (6%) | 4 (3%) | 20 (8%) |
|  | Other | 46 (12%) | 27 (20%) | 19 (8%) |
| Distance to first provider (km) | < 1 KM | 85 (22%) | 29 (21%) | 56 (23%) |
|  | 1-2 KM | 208 (55%) | 70 (51%) | 138 (57%) |
|  | 2+ KM | 85 (22%) | 39 (28%) | 46 (19%) |
|  | Don’t know | 1 (0%) | 0 (0%) | 1 (0%) |
| Method of transport | Walk | 284 (75%) | 109 (79%) | 175 (73%) |
|  | Bike | 50 (13%) | 17 (12%) | 33 (14%) |
|  | Boda (motorcycle) | 41 (11%) | 12 (9%) | 29 (12%) |
|  | Other/NA | 4 (1%) | 0 (0%) | 4 (1%) |
| How many additional people travelled? | 0 | 16 (4%) | 3 (2%) | 13 (5%) |
|  | 1 | 169 (45%) | 44 (32%) | 125 (52%) |
|  | 2 | 191 (50%) | 90 (65%) | 101 (42%) |
|  | 3 | 3 (1%) | 1 (1%) | 2 (1%) |
| Type of provider seen | Nurse | 213 (56%) | 78 (57%) | 135 (56%) |
|  | Medical Assistant | 57 (15%) | 35 (25%) | 22 (9%) |
|  | Pharmacist | 48 (13%) | 0 (0%) | 48 (20%) |
|  | Nursing Assistant | 21 (6%) | 3 (2%) | 18 (7%) |
|  | Doctor | 21 (6%) | 8 (6%) | 13 (5%) |
|  | Village health worker | 14 (4%) | 13 (9%) | 1 (0%) |
|  | Other* | 5 (1%) | 1 (1%) | 4 (2%) |
| Were you advised to have a malaria test? | Not advised to test, not tested | 124 (33%) | 18 (13%) | 106 (44%) |
|  | No advised to test, tested | 12 (3%) | 5 (4%) | 7 (3%) |
|  | Advised to test, not tested | 11 (3%) | 2 (1%) | 9 (4%) |
|  | Advised to test, tested | 232 (61%) | 113 (82%) | 119 (49%) |
| If had test, which one?- | mRDT | 217 (57%) | 106 (90%) | 111 (88%) |
|  | Microscopy | 17 (4%) | 5 (4%) | 12 (10%) |
|  | mRDT + microscopy | 9 (2%) | 6 (5%) | 3 (2%) |
|  | Don't know | 1 (0%) | 1 (1%) | 0 (0%) |
| Of those tested, result? | Positive for malaria | 208 (85%) | 96 (81%) | 112 (89%) |
|  | Negative for malaria | 34 (14%) | 21 (18%) | 13 (10%) |
|  | Don’t know | 2 (1%) | 1 (1%) | 1 (1%) |
| Did you receive prescription or medicines | Received medicine | 326 (86%) | 108 (78%) | 218 (90%) |
|  | Received prescription but no medicine | 42 (11%) | 23 (17%) | 19 (8%) |
|  | Neither | 11 (3%) | 7 (5%) | 4 (2%) |
| Received AL tablets? | Yes | 224 (59%) | 86 (62%) | 138 (57%) |
| Received DP tablets? | Yes | 8 (2%) | 0 (0%) | 8 (3%) |
| Received Artesunate IV? | Yes | 15 (4%) | 2 (1%) | 13 (5%) |
| Received Quinine IV? | Yes | 11 (3%) | 1 (1%) | 10 (4%) |
| Received Panadol tabs? | Yes | 224 (59%) | 69 (50%) | 155 (64%) |
| Received Amoxicillin tablets? | Yes | 31 (8%) | 10 (7%) | 21 (9%) |
| Other Medicines?** | Yes | 17 (5%) | 2 (1%) | 15 (6%) |
| Nights spent at facility | 0 | 350 (92%) | 125 (91%) | 225 (93%) |
|  | 1 | 9 (2%) | 5 (4%) | 4 (2%) |
|  | 2+ | 20 (5%) | 8 (6%) | 12 (5%) |
| Asked to return to provider? | Yes | 62 (16%) | 24 (17%) | 38 (16%) |
|  | No | 317 (84%) | 114 (83%) | 203 (84%) |
| Number of Additional visits | 0 | 24 (39%) | 11 (46%) | 13 (34%) |
|  | 1 | 21 (34%) | 7 (29%) | 14 (37%) |
|  | 2+ | 17 (27%) | 6(25%) | 11 (29%) |

**Other includes receiving treatment on credit, knowing a friend or relative at the clinic and qualification of staff.*

*§ Only sought treatment at public facilities*

*†Sought treatment at a private facility either for the first place of treatment or second*

***Other medicines: Artesunate, Amodiaquine*

### Supplementary Table S8: Household mean economic costs per malaria episode by cost category, costing method, type of treatment

|  | **All Suspected Cases** | **Did not seek treatment** | **Sought Treatment** | | | | | | | | | | | | | |
| --- | --- | --- | --- | --- | --- | --- | --- | --- | --- | --- | --- | --- | --- | --- | --- | --- |
|  |  |  | **Outpatient Case** | | | | | | | **Inpatient Case** | | | | | | |
|  |  |  | **All** | **Tested** | **Not tested** | **Tested Positive** | **Tested negative** | **Private facility** | **Public facility** | **All** | **Tested** | **Not tested** | **Tested Positive** | **Tested negative** | **Private facility** | **Public facility** |
|  | *n=615* | *n=235* | *n=350* | *n=219* | *n=131* | *n=189* | *n=30* | *n = 225* | *n= 125* | *n=29* | *n=24* | *n=5* | *n=21* | *n=3* | *n = 16* | *n= 13* |
| **OOP Costs (Method 1)** | 1.13 | - | 1.58 | 2.03 | 0.83 | 2.14 | 1.31 | 2.33 | 0.24 | 4.84 | 5.78 | 0.35 | 6.22 | 2.71 | 5.85 | 3.61 |
| **OOP Costs**  **(Method 2)** | |  |  |  |  |  |  |  |  |  |  |  |  |  |  |  |
| Consultation | 0.12 | - | 0.16 | 0.25 | 0.01 | 0.19 | 0.64 | 0.23 | 0.03 | 0.56 | 0.58 | 0.43 | 0.67 | - | 0.87 | 0.18 |
| Diagnostics | 0.14 | - | 0.21 | 0.34 | 0.00 | 0.34 | 0.31 | 0.32 | 0.03 | 0.38 | 0.46 | - | 0.50 | 0.18 | 0.40 | 0.35 |
| Medicines | 0.51 | - | 0.72 | 0.75 | 0.66 | 0.79 | 0.50 | 1.06 | 0.11 | 2.18 | 2.59 | 0.19 | 2.95 | 0.09 | 3.63 | 0.39 |
| Transport | 0.12 | - | 0.18 | 0.23 | 0.10 | 0.20 | 0.40 | 0.26 | 0.04 | 0.39 | 0.40 | 0.35 | 0.34 | 0.81 | 0.42 | 0.35 |
| Food | 0.08 | - | 0.11 | 0.15 | 0.03 | 0.15 | 0.17 | 0.11 | 0.11 | 0.42 | 0.51 | 0.01 | 0.58 | - | 0.22 | 0.67 |
| Total | 0.97 | - | 1.38 | 1.73 | 0.81 | 1.68 | 2.03 | 1.98 | 0.31 | 3.93 | 4.54 | 0.99 | 5.03 | 1.08 | 5.54 | 1.94 |
| **Productivity Loss** |  |  |  |  |  |  |  |  |  |  |  |  |  |  |  |  |
| due to transport | 0.37 | - | 0.58 | 0.64 | 0.47 | 0.64 | 0.60 | 0.53 | 0.65 | 0.78 | 0.87 | 0.36 | 0.91 | 0.57 | 0.83 | 0.73 |
| due to waiting | 0.48 | - | 0.80 | 1.08 | 0.32 | 1.11 | 0.94 | 0.53 | 1.27 | 0.62 | 0.71 | 0.20 | 0.71 | 0.69 | 0.75 | 0.44 |
| due to illness | 3.86 | 2.60 | 4.59 | 5.29 | 3.43 | 5.35 | 4.89 | 5.46 | 3.04 | 5.08 | 5.93 | 0.99 | 5.64 | 8.00 | 4.73 | 5.52 |
| due to caregiving | 3.87 | 1.41 | 5.10 | 5.96 | 3.67 | 6.35 | 3.51 | 5.22 | 4.90 | 8.96 | 9.32 | 7.24 | 9.71 | 6.58 | 8.84 | 9.11 |
| Total | 8.58 | 4.02 | 11.07 | 12.97 | 7.89 | 13.45 | 9.95 | 11.74 | 9.86 | 15.44 | 16.83 | 8.78 | 16.97 | 15.84 | 15.15 | 15.80 |
| **Total Economic Cost (95% CI)*** | 9.71 (8.26-11.16) | 4.02  (2.89-  5.15) | 12.65 (10.36-14.94) | 15.00 (11.50-18.50) | 8.72 (7.05-10.40) | 15.59 (11.59-19.60) | 11.26 (7.08-15.43) | 14.07 (10.63-17.51) | 10.10 (8.41-11.78) | 20.29 (14.29-26.28) | 22.61 (15.77-29.45) | 9.13 (1.00-17.26) | 23.19 (15.61-30.78) | 18.55  (-17.56 – 54.66) | 21.00 (12.47-29.53) | 19.41 (9.70-29.12) |

*We collected data on out-of-pocket (OOP) cost by asking how much household members paid out-of-pocket at a given place of care in total (Method 1), and then asking more detailed, disaggregated questions for each cost category (Method 2).*

**Total economic costs include OOP estimated from Method 1*

*All costs reported in constant USD 2022.*

### Supplementary Table S9: Treatment seeking behaviour and household economic costs per suspected case of malaria by equity-relevant variables

|  | **All household members** | | | **Suspected malaria cases** | | | | **Suspected cases that sought treatment** | | |
| --- | --- | --- | --- | --- | --- | --- | --- | --- | --- | --- |
|  | **Frequency** | | **Frequency (%)** | | | **Mean cost of illness** | | **Frequency (%)** | | **Mean cost of Illness** |
| **All** | 16 184 | | 614(4%) | | | 9.71 | | 379(62%) | | 13.24 |
| **Age of household member** | |  | | |  | |  | |  | |
| < 5 years | 3063 | | 235(8%) | | | 6.89 | | 160(68%) | | 9.49 |
| 5 – 15 years | 5543 | | 230(4%) | | | 6.59 | | 126(55%) | | 10.00 |
| 16 + years | 7578 | | 149(2%) | | | 18.96 | | 93(37%) | | 24.07 |
| **Sex of household member** | |  | | |  | |  | |  | |
| Male | 7292 | | 267(4%) | | | 8.60 | | 164(61%) | | 12.29 |
| Female | 8892 | | 347(4%) | | | 10.56 | | 215(62%) | | 13.96 |
| **Relation to household head** | |  | | |  | |  | |  | |
| Head | 3518 | | 80(2%) | | | 23.26 | | 56(70%) | | 27.25 |
| 1st degree | 10 462 | | 419(4%) | | | 7.68 | | 252(60%) | | 10.98 |
| 2nd degree | 2034 | | 106(5%) | | | 7.30 | | 66(62%) | | 10.09 |
| Not related | 170 | | 9(5%) | | | 12.14 | | 5(56%) | | 11.46 |
| **Household wealth quintile** | |  | | |  | |  | |  | |
| Poorest Q1 | 3046 | | 88(3%) | | | 6.11 | | 44(50%) | | 8.13 |
| Q2 | 3369 | | 121(4%) | | | 9.85 | | 74(61%) | | 12.96 |
| Q3 | 3261 | | 132(4%) | | | 8.67 | | 83(63%) | | 11.14 |
| Q4 | 3283 | | 149(5%) | | | 9.65 | | 95(64%) | | 13.50 |
| Least Poor Q5 | 3225 | | 124(4%) | | | 13.35 | | 83(67%) | | 18.12 |
| **Sub-region** |  | |  | | |  | |  | |  |
| North Buganda | 959 | | 4(0%) | | | 12.19 | | 4(100%) | | 12.19 |
| Bunyoro | 1793 | | 72(4%) | | | 9.20 | | 48(67%) | | 12.13 |
| West Nile | 1562 | | 35(2%) | | | 8.14 | | 23(66%) | | 6.04 |
| Acholi | 3640 | | 92(3%) | | | 9.55 | | 44(48%) | | 16.05 |
| Lango | 2001 | | 145(7%) | | | 9.74 | | 120(83%) | | 11.53 |
| Teso | 2280 | | 106(5%) | | | 11.14 | | 71(67%) | | 13.78 |
| Busoga | 2535 | | 151(6%) | | | 9.71 | | 63(42%) | | 18.37 |
| Bukedi | 480 | | 8(2%) | | | 2.57 | | 6(75%) | | 3.43 |
| Tooro | 460 | | 1(0%) | | | 4.93 | | 0(0%) | | - |
| Karamoja | 474 | | 0(0%) | | | - | | 0(0%) | | - |

*All costs reported in constant USD 2022.*

### Supplementary Figure S3 Methods for estimating household out-of-pocket costs

*We collected data on out-of-pocket (OOP) cost by asking how much household members paid out-of-pocket at a given place of care in total (Method 1), and then asking more detailed, disaggregated questions for each cost category (Method 2).*

*All costs reported in constant USD 2022.*

### Supplementary Table S10: Household mean costs per suspected case by National SES Quintile and percentage of per capita consumption

|  | **Consumption per capita*** | | **Outpatient Case** | | | **Inpatient Case** | | |
| --- | --- | --- | --- | --- | --- | --- | --- | --- |
| Uganda-wide National SES Quintile*^§^* | **Daily** | **Monthly** | **n** | **Household cost per case** | **% of monthly per capita consumption** | **n** | **Household cost per case** | **% of monthly per capita consumption** |
| Poorest Q1 | 1.26 | 37.90 | 128 | 9.92 | 26% | 10 | 23.15 | 61% |
| Q2 | 2.08 | 62.34 | 41 | 11.35 | 18% | 5 | 10.27 | 16% |
| Q3 | 2.92 | 87.49 | 61 | 14.76 | 17% | 8 | 17.56 | 20% |
| Q4 | 4.20 | 126.10 | 85 | 12.28 | 10% | 4 | 32.24 | 26% |
| Least Poor Q5 | 9.53 | 285.86 | 35 | 21.82 | 8% | 2 | 18.01 | 6% |

**Estimated from the World Bank Poverty and Inequality Platform percentiles. The average mean per capita consumption was used for each quintile.*

*§ We assigned the respondents in our study population to Uganda-wide national wealth quintile using EquityTool.*

*All costs reported in constant USD 2022.*

### Supplementary Table 11: Drivers of household cost per suspected case of malaria, alternative theory-driven model

| **Explanatory Variables** | | **Mean** |  | |  | | **Two Part Model** | | | | | |
| --- | --- | --- | --- | --- | --- | --- | --- | --- | --- | --- | --- | --- |
|  |  |  | **Logit Model**  **n=463** | | | | | **General Linearized Model**  **n=471** | | | **Marginal Effects** | |
|  |  |  | **Odds Ratio** | **p-value** | | **95% CI** | | **Odds Ratio** | **p-value** | **95% CI** | **Coefficient** | **p-value** |
| Gender | Male | 6.59 | *ref* | *ref* | | *ref* | | *ref* | *ref* | *ref* | *ref* | *ref* |
|  | Female | 7.43 | 0.91 | 0.639 | | 0.60-1.38 | | 0.98 | 0.92 | 0.80-1.22 | -0.26 | 0.751 |
| Age | <15 years | 6.74 | Omitted *§* | - | | - | | *ref* | *ref* | *ref* | *ref* | *ref* |
|  | 16+ years | 18.96 | Omitted *§* | - | | - | | 2.11 | <0.001 | 1.67-2.68 | 5.03 | <0.001 |
| Wealth | Percentile | NA | 1.01 | 0.02 | | 1.00-1.02 | | 1.01 | 0.006 | 1.00-1.01 | 0.06 | 0.001 |
| Sub-region | North Buganda | 12.19 | *ref* | *ref* | | *ref* | | *ref* | *ref* | *ref* | *ref* | *ref* |
|  | Bunyoro | 9.20 | 0.91 | 0.915 | | 0.15-5.39 | | 1.07 | 0.90 | 0.34-3.37 | 0.15 | 0.95 |
|  | West Nile | 8.14 | 1.82 | 0.539 | | 0.27-12.18 | | 1.41 | 0.57 | 0.43-4.66 | 2.45 | 0.39 |
|  | Acholi | 9.55 | 0.69 | 0.684 | | 0.12-3.97 | | 2.25 | 0.17 | 0.71-7.14 | 3.75 | 0.18 |
|  | Lango | 9.74 | 2.69 | 0.263 | | 0.47-15.21 | | 1.83 | 0.30 | 0.59-5.69 | 4.87 | 0.06 |
|  | Teso | 11.14 | 1.85 | 0.494 | | 0.31-10.89 | | 1.78 | 0.32 | 0.57-5.56 | 4.12 | 0.12 |
|  | Busoga | 9.71 | 0.50 | 0.428 | | 0.09-2.75 | | 1.68 | 0.37 | 0.54-5.21 | 1.11 | 0.65 |
|  | Bukedi | 2.57 | Omitted † | - | | - | | 0.39 | 0.19 | 0.09-1.61 | -2.34 | 0.32 |
|  | Tooro | 4.93 | Omitted † | - | | - | | 0.33 | 0.37 | 0.03-3.84 | -2.57 | 0.33 |
|  | Karamoja ** | - | - | - | | - | | - | - |  | - | - |
| Pseudo R^2^ | |  | 0.0721 |  | |  | |  |  |  |  |  |
|  | Deviance |  |  |  | |  | | 472.9 |  |  |  |  |
|  | Pearson |  |  |  | |  | | 578.7 |  |  |  |  |
|  | AIC |  |  |  | |  | | 23.38 |  |  |  |  |
|  | BIC |  |  |  | |  | | -2352 |  |  |  |  |

*Theory-based model that includes gender which was removed from the parsimonious model presented in the main paper.*

*§ Variable omitted from model because category predicted success perfectly.*

*†Omitted from model due to collinearity.*

*** No observations.*

*All costs reported in constant 2022 USD.*

## Societal Costs

### Supplementary Table S12: Disaggregated societal mean financial cost per suspected case of malaria

|  | All suspected cases  (n=614) | Untreated suspected cases  (n=235) | Treated suspected cases | | Parasitologically confirmed cases | |
| --- | --- | --- | --- | --- | --- | --- |
|  |  |  | **Outpatient**  **(n=350)** | **Inpatient**  **(n=29)** | **Outpatient**  **(n=190)** | **Inpatient**  **(n=21)** |
| Health Service Costs |  |  |  |  |  |  |
| Consultation | 1.22 | 0.00 | 1.70 | 5.39 | 2.31 | 4.67 |
| Diagnostics | 0.15 | 0.00 | 0.24 | 0.21 | 0.38 | 0.21 |
| Drugs | 0.10 | 0.00 | 0.15 | 0.25 | 0.22 | 0.30 |
| Household Costs |  |  |  |  |  |  |
| OOP costs (method 1)* | 1.13 | 0.00 | 1.58 | 4.84 | 2.14 | 6.20 |
| Total Financial Costs (95% CI) | 2.60  (1.49-2.91) | 0.00 | 3.67  (3.33-4.01) | 10.71  (7.54-13.87) | 5.04  (4.55-5.54) | 11.40  (7.27-15.52) |

** Out of pocket (OOP) costs estimated from method 1 (single question).*

*All costs reported in constant USD 2022.*

*Financial costs presented here; economic costs found in the main paper.*

### Supplementary Table S13: Sensitivity Analysis of societal cost per case of suspected malaria (a) input parameters and justification and (b) cost outputs

| **Variable Description** | **Best estimate** | **Lower estimate** | **Upper estimate** | **Justification** |
| --- | --- | --- | --- | --- |
| ***Outpatient cases*** |  |  |  |  |
| Value of one day of lost productivity | 3.29 | 2.17 | 6.70 | Lower: rural median household monthly income from the Uganda National Survey Report 2019-2022 (UBOS, 2021)  Higher: GDP per adult (World Bank) |
| Reported duration of days lost work | 3 | 1 | 5 | +/- SD (1.95 days) |
| Reported duration of caregiver days | 1.5 | 0 | 3.5 | +/- SD (2.04 days) |
| Productivity loss if sick and reported to work | .50 | 0 | .75 | Current cost-of-illness publications inform the ranges (Hansen & Yeung, 2019) |
| Test cost for RDT | 0.60 | 0.30 | 0.90 | +/-50%: Based on range of costs found in The Global Fund Pooled Procurement Mechanism Reference Pricing: RDTs (The Global Fund, 2022) |
| Cost of AL | 0.38 | 0.19 | 0.57 | +/-50%: Based on range of costs found in The Global Fund Pooled Procurement Mechanism Reference Pricing: Antimalarial medicines (The Global Fund, 2022) |
| Using Method 1 for OOP | 1.58 | 1.38 | - | Methods informed by ACT consortium guide on collecting household costs (Hansen &Yeung, 2019) and Agorinya et al, 2021 |
| Discount rate for health service costs | 0.03 | 0.01 | 0.05 | Current cost-of-illness publications inform the ranges (Hansen & Yeung, 2019) |
| Valuation of sq meter at government run health centres | 200.00 | 140.00 | 260 | +/-30%: Based on conversations with local construction experts |
| Consultation cost at government run health centres | 4.91 | 3.44 | 6.38 | +/-30%: Authors assumption |
| ***Inpatient cases*** |  |  |  |  |
| Value of one day of lost productivity | 3.29 | 2.17 | 6.70 | Lower: rural median household monthly income from the Uganda National Survey Report 2019-2022 (UBOS, 2021)  Higher: GDP per adult (World Bank) |
| Reported duration of days lost work | 3.5 | 1.5 | 5.5 | +/- SD (1.95 days) |
| Reported duration of caregiver days | 2.70 | 0.4 | 5 | +/- SD (2.3 days) |
| Productivity loss if sick and reported to work | 50 | 0 | 75 | Current cost-of-illness publications inform the ranges (Hansen & Yeung, 2019) |
| Test cost for RDT | 0.60 | 0.30 | 75.00 | +/-50%: Based on range of costs found in The Global Fund Pooled Procurement Mechanism Reference Pricing: RDTs (The Global Fund, 2022) |
| Cost of AL | 0.38 | 0.00 | 0.61 | +/-50%: Based on range of costs found in The Global Fund Pooled Procurement Mechanism Reference Pricing: Antimalarial medicines (The Global Fund, 2022) |
| Using Method 1 for OOP | 4.84 | 3.93 | - | Methods informed by ACT consortium guide on collecting household costs (Hansen &Yeung, 2019) and Agorinya et al, 2021 |
| Discount rate for health service costs | 3 | 1 | 5 | Current cost-of-illness publications inform the ranges (Hansen & Yeung, 2019) |
| Valuation of meter^2^ at government run health centres | 200.00 | 140.00 | 260.00 | +/-30%: Based on conversations with local construction experts |
| Consultation cost at government run health centres | 12.37 | 8.66 | 16.08 | +/-30%: Authors assumption |

Agorinya, Isaiah Awintuen, Ross, Amanda, Flores, Gabriela, Tantorres Edejer, Tessa, Dalaba, Maxwell Ayindenaba, Mensah, Nathan Kumasenu, et al., ‘Effect of Specificity of Health Expenditure Questions in the Measurement of Out-of-Pocket Health Expenditure: Evidence from Field Experimental Study in Ghana’, *BMJ Open*, 11/5 (2021)

Hansen, Kristian Schultz, and Yeung, Shunmay, ‘ACT Consortium Guidance on Collecting Household Costs.’, 2009, 1–11 <http://www.actconsortium.org/data/files/household_costs.pdf>

The Global Fund, *Pooled Procurement Mechanism Reference Pricing : Antimalarial Medicines*, 2022 <https://www.theglobalfund.org/media/5812/ppm_actreferencepricing_table_en.pdf>

———, *Pooled Procurement Mechanism Reference Pricing : RDTs*, 2022 <https://www.theglobalfund.org/media/7564/psm_hivrdtreferencepricing_table_en.pdf>

Ugandan Bureau of Statistics, *Uganda National Survey Report 2019-2020*, 2021 <https://www.ubos.org/wp-content/uploads/publications/09_2021Uganda-National-Survey-Report-2019-2020.pdf>

World Bank, ‘GDP (Current US$) - Uganda’, 2023 <https://data.worldbank.org/indicator/NY.GDP.MKTP.CD?locations=UG>

|  | **Lower Estimate** | | **Upper Estimate** | |
| --- | --- | --- | --- | --- |
| *Variable Description* | **Cost per case** | **% change** | **Cost per case** | **% change** |
| ***Outpatient cases*** |  |  |  |  |
| *Value of one day of lost productivity* | 11.34 | 25% | 26.59 | -76% |
| *Reported duration of days lost work* | 14.01 | 7% | 16.63 | -10% |
| *Reported duration of caregiver days* | 12.07 | 20% | 18.39 | -22% |
| *Productivity loss if sick and reported to work* | 14.52 | 4% | 15.42 | -2% |
| *Test cost for RDT* | 14.99 | 1% | 15.20 | -1% |
| *Cost of AL* | 15.04 | 1% | 15.14 | 0% |
| *Using Method 1 for OOP* | 14.93 | 1% | - | - |
| *Discount rate for health service costs* | 15.07 | 0% | 15.16 | 0% |
| *Valuation of sq meter at government run health centres* | 15.10 | 0% | 15.14 | 0% |
| *Consultation cost at government run health centres* | 14.57 | 4% | 15.87 | -5% |
| ***Inpatient cases*** |  |  |  |  |
| *Value of one day of lost productivity* | 21.94 | 19% | 43.21 | -59% |
| *Reported duration of days lost work* | 25.70 | 6% | 29.39 | -8% |
| *Reported duration of caregiver days* | 22.56 | 17% | 32.20 | -18% |
| *Productivity loss if sick and reported to work (0-75%)* | 26.89 | 1% | 27.37 | -1% |
| *Test cost for RDT* | 27.10 | 0% | 27.29 | 0% |
| *Cost of AL* | 27.15 | 0% | 27.22 | 0% |
| *Using Method 1 for OOP* | 26.30 | 3% | - | - |
| *Discount rate for health service costs* | 27.19 | 0% | 27.23 | 0% |
| *Valuation of sq meter at government run health centres* | 27.17 | 0% | 27.25 | 0% |
| *Consultation cost at government run health centres* | 27.15 | 0% | 27.36 | -1% |

### Supplementary Figure S4: Deterministic sensitivity analysis for societal costs; tornado diagram of (a) treated outpatient suspected malaria and (b) treated inpatient suspected malaria

(a)

(b)

### Supplementary Table S14: Economic burden of malaria in Uganda

|  | | **Financial Costs** | | | **Economic Costs** | | |
| --- | --- | --- | --- | --- | --- | --- | --- |
| ***Perspective*** | | ***Health Service*** | ***Household*** | ***Societal*** | ***Health Service*** | ***Household*** | ***Societal*** |
| **Burden due to morbidity** | |  |  |  |  |  |  |
| Untreated suspected malaria cases | Number of cases, 2021 | 1 693 042 | | | | | |
|  | Cost per case | 0 | 4.02 | 4.02 | 0 | 4.02 | 4.02 |
|  | Total costs | 0 | 6 806 027 | 6 806 027 | 0 | 6 806 027 | 6 806 027 |
| Outpatient case | Number of cases, 2021 | 11 037 329 | | | | | |
|  | Cost per case | 2.09 | 1.58 | 3.67 | 2.47 | 12.65 | 15.12 |
|  | Total costs | 23 068 018 | 17 438 980 | 40 506 997 | 27 262 203 | 139 622 211 | 166 884 414 |
| Inpatient cases | Number of cases, 2021 | 293 026 | | | | | |
|  | Cost per case | 5.86 | 4.84 | 10.7 | 6.92 | 20.29 | 27.21 |
|  | Total costs | 1 717 135 | 1 418 248 | 3 135 383 | 2 027 743 | 5 945 506 | 7 973 249 |
| All cases | Total costs | 24 785 152 | 25 663 255 | 50 448 407 | 29 289 945 | 152 373 745 | 181 663 690 |
|  | Cost per capita | 0.54 | 0.56 | 1.10 | 0.64 | 3.32 | 3.96 |
| **Burden due to mortality** | |  |  |  |  |  |  |
|  | Number of deaths, 2021 | 21 699 | | | | | |
|  | Cost per lost life | 0 | 0 | 0 | 0 | 18 199 | 18 199 |
|  | Total costs | 0 | 0 | 0 | 0 | 394 907 110 | 394 907 110 |
| **Total burden** | |  |  |  |  |  |  |
| Base case estimate | Total costs | 24 785 152 | 25 663 255 | 50 448 407 | 29 289 945 | 547 280 855 | 576 570 801 |
|  | Cost per capita | 0.54 | 0.56 | 1.10 | 0.64 | 11.94 | 12.57 |
| Scenario analysis: Best case estimate | Morbidity costs | 13 545 234 | 13 149 919 | 26 695 153 | 15 974 909 | 79 777 723 | 95 752 632 |
|  | Mortality costs | 0 | 0 | 0 | 0 | 206 383 095 | 206 383 095 |
|  | All costs | 13 545 234 | 13 149 919 | 26 695 153 | 15 974 909 | 286 160 818 | 302 135 727 |
|  | Cost per capita | 0.30 | 0.29 | 0.58 | 0.35 | 6.24 | 6.59 |
| Scenario analysis: Worst case estimate | Morbidity costs | 47,426,083 | 51,094,663 | 98,520,746 | 56,128,450 | 294,663,643 | 350,792,093 |
|  | Mortality costs | 0 | 0 | 0 | 0 | 738 456 679 | 738,456,679.17 |
|  | All costs | 47 426 083 | 51 925 658 | 98,520,746 | 54 941 314 | 1 033 951 317 | 1,089,248,772 |
|  | Cost per capita | 1.03 | 1.11 | 2.15 | 1.22 | 22.55 | 23.75 |

All costs reported in constant USD 2022.
