# Supplementary material for: Who pays to treat malaria and how much? Analysis of the cost of illness, equity and economic burden of malaria in Uganda
Source: Health Policy Plan. 2024 Oct 15;40(1):52–65. doi: 10.1093/heapol/czae093 (PMC11724642; doi:10.1093/heapol/czae093)
Supplement: czae093_Supp [file czae093_supp.zip › czae093_Supp/COI Table 3.docx]

|  | All suspected cases  (n=614) | Untreated suspected cases  (n=235) | Treated suspected cases | | Parasitologically confirmed cases | |
| --- | --- | --- | --- | --- | --- | --- |
|  |  |  | **Outpatient**  (n=350) | **Inpatient**  (n=29) | **Outpatient**  (n=190) | **Inpatient**  (n=21) |
| Health Service Costs |  |  |  |  |  |  |
| Consultation | 1.49 | 0.00 | 2.08 | 6.46 | 2.82 | 5.59 |
| Diagnostics | 0.15 | 0.00 | 0.24 | 0.21 | 0.38 | 0.21 |
| Drugs | 0.10 | 0.00 | 0.15 | 0.25 | 0.22 | 0.30 |
| Total Health Service costs  (95% CI) | 1.73  (1.49-1.97) | 0.00  N/A | 2.47  (2.17-2.77) | 6.92  (4.49-9.36) | 3.42  (3.01-3.84) | 6.10  (3.10-9.10) |
| Household Costs |  |  |  |  |  |  |
| OOP costs (method 1)* | 1.13 | 0.00 | 1.58 | 4.84 | 2.14 | 6.22 |
| Lost time due to transport | 0.37 | 0.00 | 0.58 | 0.78 | 0.64 | 0.91 |
| Lost time due to waiting | 0.48 | 0.00 | 0.80 | 0.62 | 1.11 | 0.71 |
| Lost productivity due to illness | 3.86 | 2.60 | 4.59 | 5.08 | 5.35 | 5.64 |
| Lost productivity due to caregiving | 3.87 | 1.41 | 5.10 | 8.96 | 6.35 | 9.71 |
| Total household costs  (95% CI) | 9.71  (8.26-11.16) | 4.02  (2.89-5.15) | 12.65  (10.36-14.94) | 20.29  (14.29-26.28) | 15.59  (11.58-19.60) | 23.19  (15.61-30.78) |
| Total Societal Costs (95% CI) | 11.44  (9.95-12.94) | 4.02  (2.89-5.15) | 15.12  (12.83-17.41) | 27.21  (20.43-33.99) | 19.02  (15.06-22.98) | 29.29  (20.57-38.00) |
