# Supplementary material for: Who pays to treat malaria and how much? Analysis of the cost of illness, equity and economic burden of malaria in Uganda
Source: Health Policy Plan. 2024 Oct 15;40(1):52–65. doi: 10.1093/heapol/czae093 (PMC11724642; doi:10.1093/heapol/czae093)
Supplement: czae093_Supp [file czae093_supp.zip › czae093_Supp/COI Table1_V2.docx]

| **Health centre number** | **1** | **2** | **3** | **4** | **5** | **6** | **7** | **8** | **Total** |
| --- | --- | --- | --- | --- | --- | --- | --- | --- | --- |
| **Characteristics of Health Centres** |  |  |  |  |  |  |  |  |  |
| District | Kapelebyong | Kaabong | Oyam | Nwoya | Moyo | Amuru | Mubende | Mayuge |  |
| Sub-region | Teso | Karamoja | Lango | Acholi | West Nile | Acholi | Nort Buganda | Busoga |  |
| Region | East | North | North | North | North | North | Central | East |  |
| Health centre level * | III | III | III | III | III | IV | III | IV |  |
| Total outpatient malaria cases § | 7605 | 6584 | 9099 | 6355 | 5954 | 11 237 | 2328 | 8316 | 57 478 |
| Total inpatient malaria admissions (% total admissions) § | 576 (47%) | 143 (15%) | 519 (23%) | 26 (35%) | 362 (74%) | 1063 (61%) | 82 (47%) | 486 (60%) | 3257 (45%) |
| Total inpatient nights for malaria (% of total nights) § | 1416 (59%) | 358 (22%) | 970 (15%) | 89 (57%) | 530 (28%) | 2765 (39%) | 259 (47%) | 2081 (25%) | 859 (34%) |
| Mean nights per malaria admission | 2.5 | 2.5 | 1.9 | 3.4 | 1.5 | 2.6 | 3.2 | 1 | 2.3 |
| Total Rapid Diagnostic Tests (RDTs) performed | 11 019 | 11 735 | 8608 | 11 137 | 6717 | 15 150 | 3507 | 10 671 | 78 544 |
| Total microscopy performed | 5322 | 239 | 4941 | 1376 | 1662 | 282 | 2062 | 3729 | 19 613 |
| **Time & Motion Observations** |  |  |  |  |  |  |  |  |  |
| Number of malaria outpatient consultations observed | 17 | 17 | 27 | 7 | 19 | 10 | 17 | 12 | 126 |
| Number of non-malaria outpatient consultations observed | 12 | 42 | 14 | 25 | 20 | 7 | 22 | 11 | 153 |
| Mean malaria consultation time (minutes) | 3.5 | 3.5 | 3.8 | 10.2 | 2.7 | 4.1 | 3.2 | 3.1 | 3.8 |
| Mean non-malaria consultation time (minutes) | 2.9 | 3.1 | 3.7 | 5.8 | 3 | 3.6 | 2.9 | 3.4 | 3.6 |
