# Supplementary material for: Who pays to treat malaria and how much? Analysis of the cost of illness, equity and economic burden of malaria in Uganda
Source: Health Policy Plan. 2024 Oct 15;40(1):52–65. doi: 10.1093/heapol/czae093 (PMC11724642; doi:10.1093/heapol/czae093)
Supplement: czae093_Supp [file czae093_supp.zip › czae093_Supp/COI Table2_V2.docx]

| **Cost category** | | **Outpatient cases** | | **Inpatient cases** | |
| --- | --- | --- | --- | --- | --- |
| **Consultation & care costs** | | **Financial (range†)** | **Economic (range†)** | **Financial (range †)** | **Economic (range†)** |
| Recurrent costs | Labour | 3.49 (2.49 – 3.99) | 4.37 (3.46 – 5.27) | 8.22 (2.49 – 13.30) | 10.22 (3.75 – 16.67) |
|  | Overheads* | 0.24 (0.11 – 0.39) | 0.24 (0.11 – 0.39) | 0.53 (0.24 – 0.90) | 0.53 (0.24 – 0.90) |
| Capital costs§ | Building cost | 0.08 (0.03 – 0.13) | 0.08 (0.03 – 0.13) | 0.37 (0.12 – 0.89) | 0.37 (0.12 – 0.89) |
|  | Equipment & Furniture | 0.10 (0.05 – 0.25) | 0.13 (0.05 – 0.28) | 0.97 (0.01 – 1.75) | 1.00 (0.06 – 2.15) |
|  | Vehicle cost | 0.10 (0.00 – 0.75) | 0.10 (0.00 – 0.75) | 0.25 (0.00 – 1.95) | 0.25 (0.00 – 1.95) |
| Consultation & care cost per case | | 4.01 (2.87 – 5.22) | 4.91 (3.69 – 5.63) | 10.34 (4.74 – 15.14) | 12.37 (6.19 – 18.27) |
| **Consumable costs** | |  |  |  |  |
| Diagnostics | | 1.03 (0.80 – 1.27) | 1.06 (0.80 – 1.27) | 1.03 (0.80 – 1.27) | 1.06 (0.80 – 1.27) |
| Treatment | Medicines | 0.81 (0.65 – 0.99) | 0.81 (0.65 – 0.99) | 5.45 (N/A) | 5.45 (N/A) |
|  | Other treatment supplies | NA | NA | 2.95 (N/A) | 2.95 (N/A) |
| Consumable cost per case | | 1.83 (1.49 – 2.20) | 1.87 (1.49 – 2.20) | 9.43 (9.20 – 9.68) | 9.46 (9.20 – 9.68) |
| Total cost per case treated | | 5.84 (4.86 – 6.85) | 6.78 (5.80 – 7.83) | 19.77 (14.14 – 24.60) | 21.84 (15.59 – 27.95) |
