# Supplementary material for: Who pays to treat malaria and how much? Analysis of the cost of illness, equity and economic burden of malaria in Uganda
Source: Health Policy Plan. 2024 Oct 15;40(1):52–65. doi: 10.1093/heapol/czae093 (PMC11724642; doi:10.1093/heapol/czae093)
Supplement: czae093_Supp [file czae093_supp.zip › czae093_Supp/COI Table4_V2.docx]

| **Explanatory Variables** | | **Mean** |  | |  | | **Two Part Model** | | | | | |
| --- | --- | --- | --- | --- | --- | --- | --- | --- | --- | --- | --- | --- |
|  |  |  | **Logit Model**  **n=463** | | | | | **General Linearized Model**  **n=471** | | | **Marginal Effects** | |
|  |  |  | **Odds Ratio** | **p-value** | | **95% CI** | | **Odds Ratio** | **p-value** | **95% CI** | **Coefficient** | **p-value** |
| Age | <15 years | 6.74 | Omitted *§* | - | | - | | *ref* | *ref* | *ref* | *ref* | *ref* |
|  | 16+ years | 18.96 | Omitted *§* | - | | - | | 2.11 | <0.001 | 1.67-2.66 | 4.99 | <0.001 |
| Wealth | Percentile | NA | 1.01 | 0.01 | | 1.00-1.02 | | 1.01 | 0.006 | 1.00-1.01 | 0.06 | 0.001 |
| Sub-region | North Buganda | 12.19 | *ref* | *ref* | | *ref* | | *ref* | *ref* | *ref* | *ref* | *ref* |
|  | Bunyoro | 9.20 | 0.39 | 0.92 | | 0.15-5.41 | | 1.08 | 0.88 | 0.34-3.07 | 0.18 | 0.94 |
|  | West Nile | 8.14 | 1.82 | 0.59 | | 0.27-12.2 | | 1.41 | 0.57 | 0.43-4.65 | 2.45 | 0.38 |
|  | Acholi | 9.55 | 0.70 | 0.69 | | 0.12-4.01 | | 2.26 | 0.17 | 0.71-7.14 | 3.78 | 0.17 |
|  | Lango | 9.74 | 2.70 | 0.26 | | 0.47-15.2 | | 1.83 | 0.30 | 0.59-5.69 | 4.89 | 0.56 |
|  | Teso | 11.14 | 1.86 | 0.49 | | 0.32-10.9 | | 1.78 | 0.32 | 0.57-5.56 | 4.13 | 0.11 |
|  | Busoga | 9.71 | 0.51 | 0.43 | | 0.09-2.77 | | 1.69 | 0.36 | 0.54-5.21 | 1.14 | 0.65 |
|  | Bukedi | 2.57 | Omitted † | - | | - | | 0.39 | 0.19 | 0.09-1.61 | -2.33 | 0.32 |
|  | Tooro | 4.93 | Omitted † | - | | - | | 0.33 | 0.37 | 0.02-3.82 | -2.56 | 0.33 |
|  | Karamoja ** | - | - | - | | - | | - | - |  | - | - |
| Pseudo R^2^ | |  | 0.0717 |  | |  | |  |  |  |  |  |
|  | Deviance |  |  |  | |  | | 472.9 |  |  |  |  |
|  | Pearson |  |  |  | |  | | 577.8 |  |  |  |  |
|  | AIC |  |  |  | |  | | 23.37 |  |  |  |  |
|  | BIC |  |  |  | |  | | -2358 |  |  |  |  |
