# Supplementary figures and images for: Who pays to treat malaria and how much? Analysis of the cost of illness, equity and economic burden of malaria in Uganda
Source: Health Policy Plan. 2024 Oct 15;40(1):52–65. doi: 10.1093/heapol/czae093 (PMC11724642; doi:10.1093/heapol/czae093)

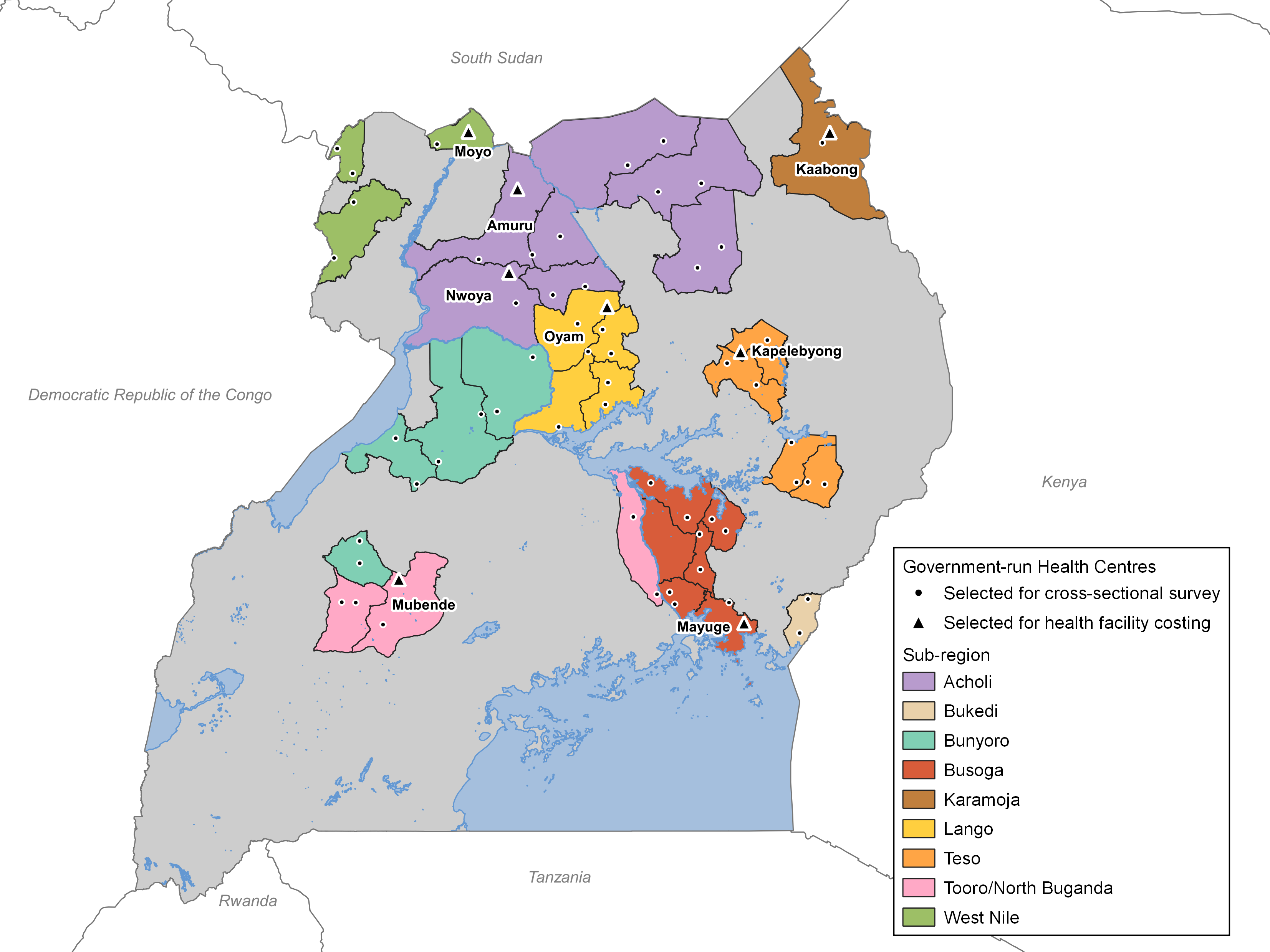

Supplement: czae093_Supp [file czae093_supp.zip › czae093_Supp/Fig1_COI.tif]

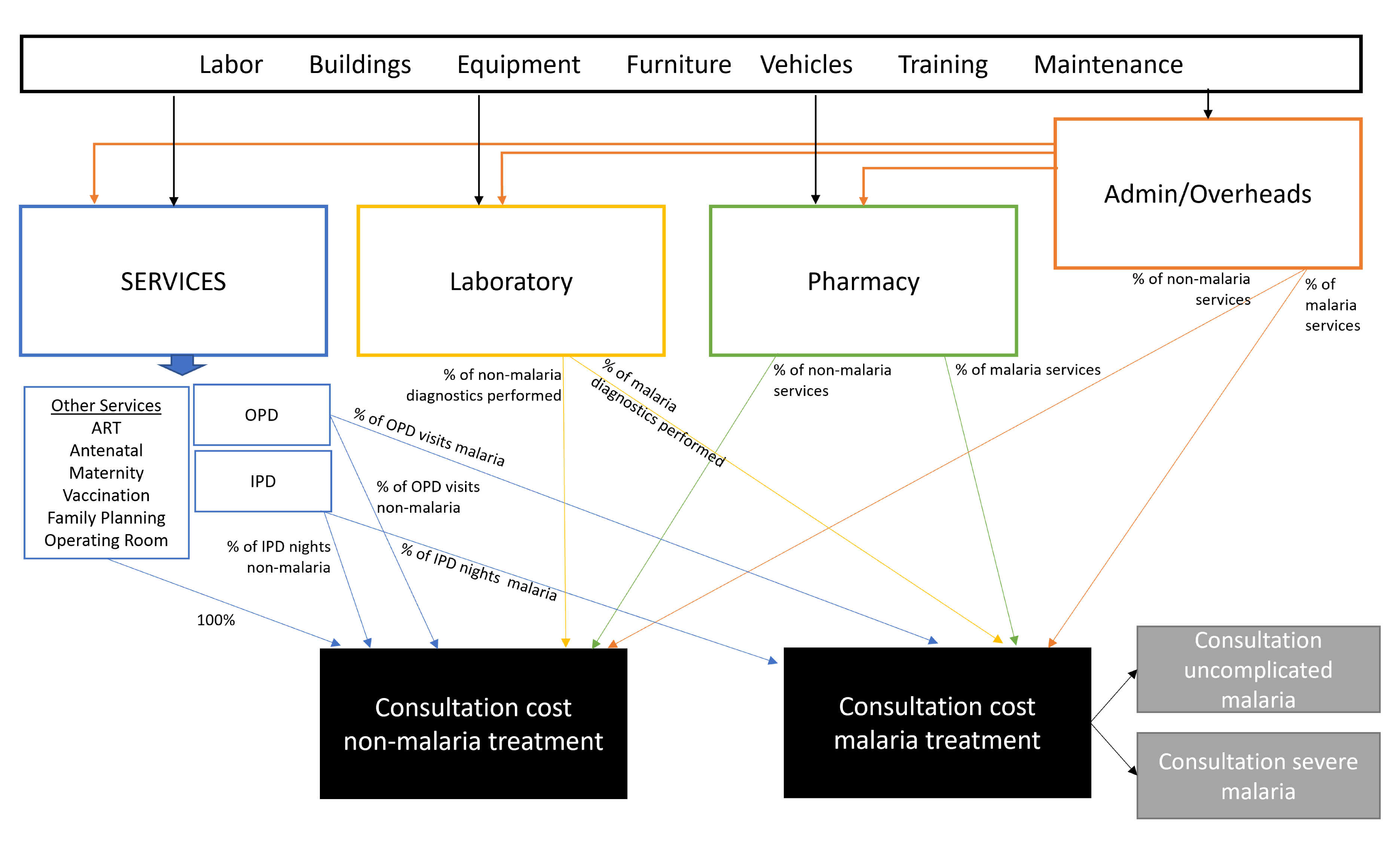

Supplement: czae093_Supp [file czae093_supp.zip › czae093_Supp/Fig2_COI.tif]

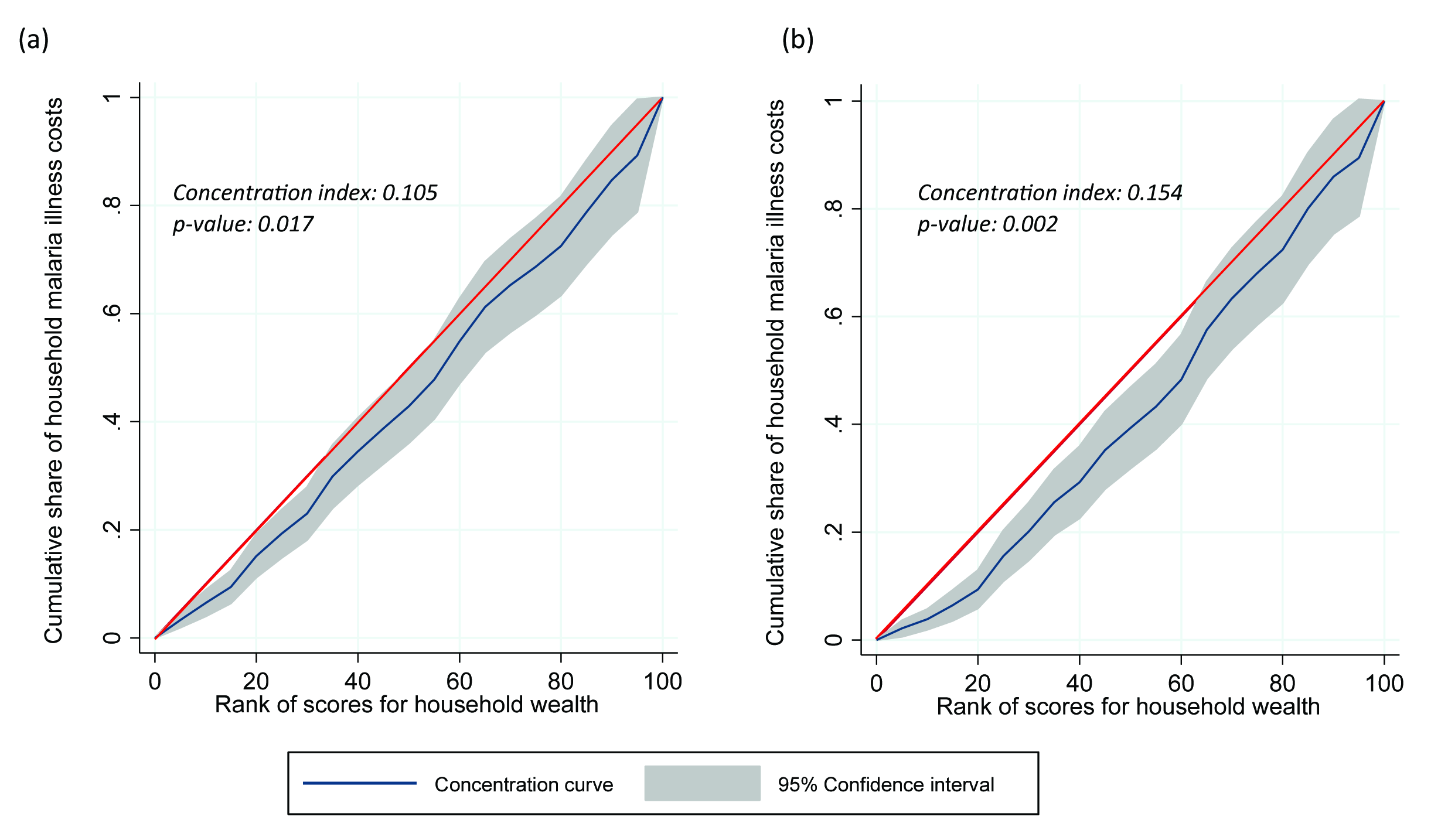

Supplement: czae093_Supp [file czae093_supp.zip › czae093_Supp/Fig3_COI.tif]
